# Is diet related to osteoarthritis? A univariable and multivariable Mendelian randomization study that investigates 45 dietary habits and osteoarthritis

**DOI:** 10.3389/fnut.2023.1278079

**Published:** 2023-11-16

**Authors:** Zhuoting Xie, Yanguo Qin

**Affiliations:** Department of Orthopedics, The Second Hospital of Jilin University, Changchun, China

**Keywords:** diet, dietary intake, osteoarthritis, total joint replacement, Mendelian randomization

## Abstract

**Background:**

Diet is a safe intervention for many chronic diseases as a modifiable lifestyle. However, the potential causal effect of many dietary intake habits on the risk of osteoarthritis has not been fully understood. The purpose of this study was to reveal the potential causal relationship of 45 genetically predicted dietary intakes with osteoarthritis and its subtypes.

**Methods:**

Data on 45 dietary intakes were obtained from the UK Biobank study of approximately 500,000 participants, and data on six osteoarthritis-related phenotypes were obtained from the Genetics of Osteoarthritis Consortium study of 826,690 participants. We performed univariable Mendelian randomization (MR), multivariable MR and linkage disequilibrium score regression (LDSC) analyses.

**Results:**

In univariate analyses, 59 potential associations between diet and osteoarthritis were found. After false discovery rate (FDR) correction and sensitivity analyses, 23 reliable causal evidence were identified. In multivariate analyses, controlling separately for the effects of body mass index, total body bone mineral density, and smoking status, eight robust causal relationships remained: Muesli intake was negatively associated with knee osteoarthritis, spine osteoarthritis and total knee replacement. Dried fruit intake had a negative association with osteoarthritis of knee and total knee replacement. Eating cheese may reduce the risk of osteoarthritis in the knee and spine. And alcohol usually taken with meals was associated with a reduced risk of total knee replacement. LDSC analyses showed significant genetic correlations between all exposures and their corresponding outcomes, respectively, in these eight causal relationships.

**Conclusion:**

Evidence of dietary effects on osteoarthritis is provided in our study, which has important implications for the prevention, management, and intervention of osteoarthritis in common sites through rational dietary modification.

## Introduction

Osteoarthritis (OA) is the most common type of arthritis, which can affect one or more different joints throughout the body (1, 2). With the aging of the population and rising rates of obesity in our society, the incidence of osteoarthritis is increasing year by year and has become a major cause of disability in adults. It has a serious impact on the quality of life of patients and places a heavy burden on society (3). The current mainstay of treatment for osteoarthritis is pain management and total joint replacement surgery at the end stage of osteoarthritis. However, these management options do not stop the progression of the disease and carry the risk of medication side effects and surgical complications. Therefore, early prevention of osteoarthritis is crucial and finding modifiable risk factors other than obesity for early intervention in osteoarthritis is urgent.

Several previous studies have suggested that dietary intake may play an important role in knee OA (4, 5). One study found a significant negative correlation between whole grain consumption and the risk of knee OA (6); whereas, current findings are inconsistent regarding the effect of consumption of dairy products such as milk and cheese on knee OA (7, 8). The most significant limitation of these studies is that they may be affected by other confounding factors that can modify lifestyle, leading to uncertainty in the results. Furthermore, few studies have emphasized the effect of subcategorization of a particular food or drink on osteoarthritis. Moreover, most of the current studies have focused on knee OA, which is the most common form of osteoarthritis, with very little research analyzing the effect of diet on osteoarthritis in other parts of the body and end-stage osteoarthritis.

In this situation, Mendelian randomization (MR) analyses provide us with a suitable method to investigate the causal relationship between specific dietary intake and osteoarthritis, utilizing single nucleotide polymorphisms as instrumental variables (IVs), which are less susceptible to confounding factors because gametes are randomly assigned during gamete formation and are independent of environmental factors (9). Previous MR analyses have explored the causal effects of coffee consumption, tea, and alcohol intake on osteoarthritis of the hip and knee (10–12). However, these studies did not classify drinks in detail and only analyzed the effects of drinks on the most common types of osteoarthritis of the hip and knee. Causal associations of other dietary intake on the risk and severity of osteoarthritis at different sites remain unknown.

In this study, we performed comprehensive univariate MR, multivariate MR and LDSC analyses using the largest known Genome wide association study (GWAS) data on diet and osteoarthritis, and thereby explored the causal relationship between 45 dietary intake habits and six osteoarthritis phenotypes.

## Materials and methods

**Figure 1:**
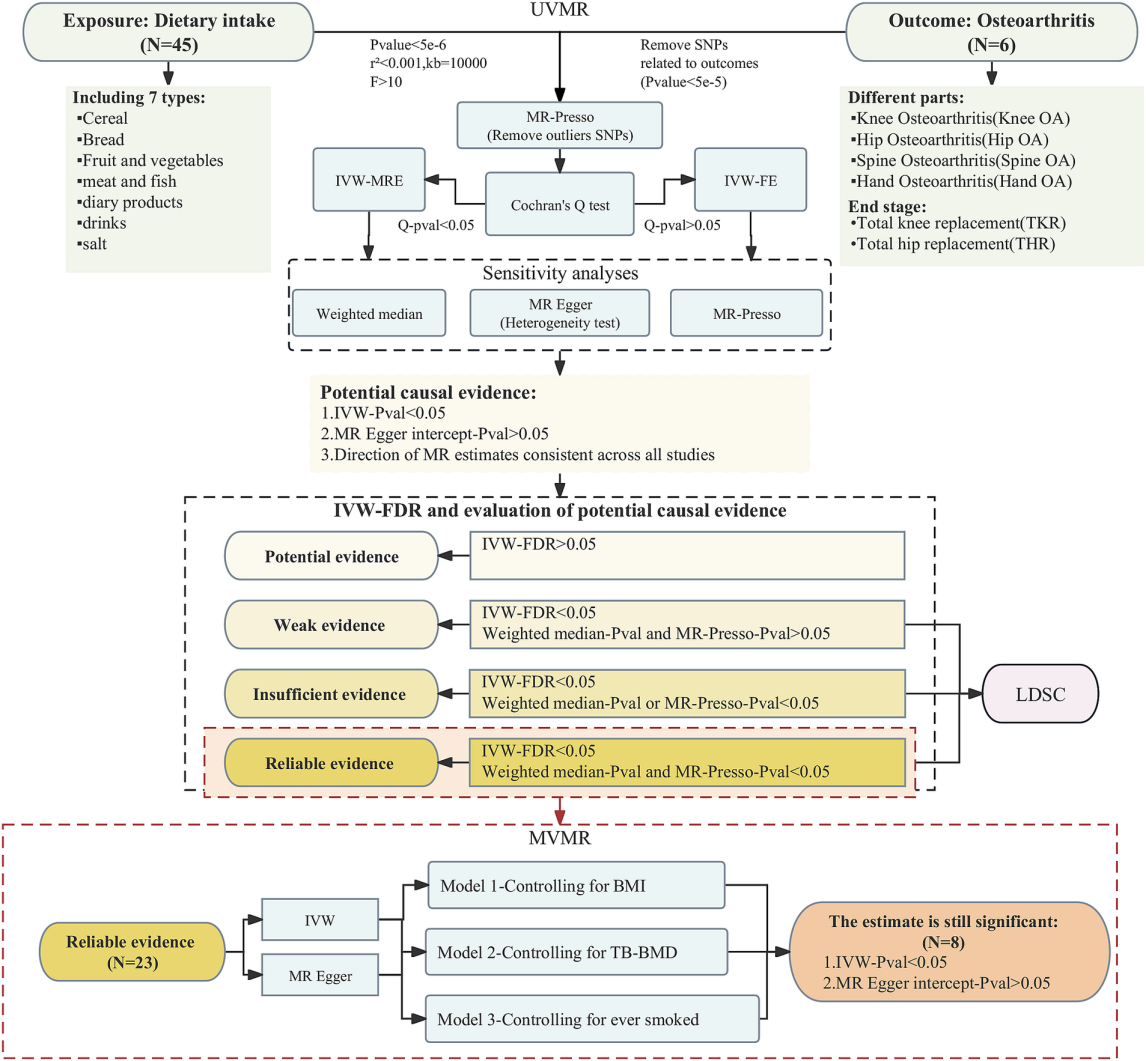
The flowchart of study design and analytic approach.

The flowchart of study design and analytic approach is shown in Figure 1.

### Data source

We obtained summary-level data related to dietary intake habits through the IEU Open GWAS program from the UK Biobank study of approximately 500,000 participants, who were recruited at ages 40–69 years across England, Wales, and Scotland from 2006 to 2010 (13). The participants provided information on lifestyle and physical measurements, donated biomedical samples, and agreed to track their health status. This study used a touchscreen questionnaire to obtain information on participants’ frequency of intake of common foods and drinks over the last year. As an example, to investigate cereal intake and the main type of cereals eaten, participants were asked, “How many bowls of cereal do you eat a WEEK? What type of cereal do you mainly eat? If you eat more than one type of cereal, please select the one that you eat the most. And please provide an average considering your intake over the last year.” Detailed information on the 45 dietary intake habits is provided in Supplementary Table S1. In addition, we obtained data on body mass index (BMI), total body bone mineral density (TB-BMD) and smoking status in the same way that was used to exclude the effects of these 3 common risk factors for osteoarthritis in the multivariate MR study.

Data on osteoarthritis were from the largest meta-analysis of genome-wide association studies on osteoarthritis to date, which included 13 cohorts with up to 826,690 participants (14). Osteoarthritis was defined by either: self-reported osteoarthritis, clinically diagnosed, ICD10 codes or radiographic depending on the data available in the cohort. Controls were osteoarthritis-free or population-based with or without ICD code exclusions. We selected osteoarthritis phenotypes from different parts of the body, including osteoarthritis of the knee (Knee OA), osteoarthritis of the hip (Hip OA), osteoarthritis of the spine (Spine OA) and osteoarthritis of the hand (Hand OA). Also, since osteoarthritis progression and osteoarthritis development were two distinct etiological endpoints, we chose total knee replacement (TKR) and total hip replacement (THR), which were associated with severe osteoarthritis, to represent end-stage knee and hip OA as a way of exploring whether dietary factors are associated with osteoarthritis progression. Details of the data sources used in this study are shown in Table 1.

**Table 1:** Description of GWAS data sources for each phenotype.

Dataset type	Variable	GWAS ID	Sample size	Consortium	Journal	Population	Sex
Exposure	Dietary habits	See Supplementary Table S1		MRC-IEU	Nature	European	Males and females
	Body mass index (BMI)	ukb-b-19953	461,460	MRC-IEU	Nature	European	Males and females
	Total body bone mineral density	ebi-a-GCST005348	56,284	NA	Am J Hum Genet.	European	Males and females
	Ever smoked	ukb-b-20261	461,066	MRC-IEU	Nature	European	Males and females
Outcome	Knee osteoarthritis (Knee OA)	NA	396,054	Genetics of Osteoarthritis (GO)	Cell	European (99%)	Males and females
	Hip osteoarthritis (Hip OA)	NA	353,388	Genetics of Osteoarthritis (GO)	Cell	European	Males and females
	Spine osteoarthritis (Spine OA)	NA	333,950	Genetics of Osteoarthritis (GO)	Cell	European (99%)	Males and females
	Hand osteoarthritis (Hand OA)	NA	303,782	Genetics of Osteoarthritis (GO)	Cell	European	Males and females
	Total knee replacement (TKR)	NA	252,041	Genetics of Osteoarthritis (GO)	Cell	European	Males and females
	Total hip replacement (THR)	NA	319,037	Genetics of Osteoarthritis (GO)	Cell	European	Males and females

### Selection of instrumental variables

In order to include more SNPs as IVs to investigate more relationships between dietary intake habits and osteoarthritis, we used the threshold of *p* < 5e-6 for IV filtering, and we removed chained unbalanced IVs (clumping: *r*^2^ = 0.001, kb = 10,000) to ensure that each IV was independent of each other (15). We also excluded SNPs associated with outcome (*p* < 5e-5) and retained only strong instrumental variables (F statistics>10) (16). Finally, we dropped outliers using MR-Presso and used the remaining SNPs as the final IVs for the MR analyses.

### Statistical analysis

In the univariate analyses, we mainly used the inverse-variance weighted (IVW) method for estimation. Heterogeneity was firstly tested for using the Cochran Q analysis, and if there was no heterogeneity, fixed-effects IVW (IVW-FE) model was used for the main analyses; otherwise, random-effects IVW (IVW-MRE) model was applied (17). In order to test the robustness and reliability of the results, we also carried out sensitivity analyses for each causal relationship using weighted median, MR Egger, and MR-Presso, and tested the results for pleiotropy using MR Egger intercept. If the results of IVW were statistically significant without pleiotropy and the estimates of the four MR analyses were in the same direction, we considered a potential causal association between the exposure and the outcome. If any other conditions existed, then we considered that no association between the exposure and the outcome was found through our study. After adjusting for *p*-values using the FDR approach, further evaluation of the strength of each potential causality evidence was performed based on the following criteria (18): (a) whether the FDR result was <0.05; (b) whether the estimates of weighted median and MR Presso were statistically significant. The degree of evidence strength for each causal relationship is classified as reliable, insufficient, weak, or potential. We screened for reliable evidence of causality for further multivariate MR analyses to explore whether the screened dietary intakes were still associated with outcomes after excluding BMI, TB-BMD and smoking status, independently. Three common risk factors for osteoarthritis were considered as multivariate models: (a) M1: BMI; (b) M2: TB-BMD (c) M3: ever smoked. In the multivariate MR analyses, we mainly applied the IVW method and the MR Egger method was also used to determine whether the results were pleiotropic or not. The statistical analyses in this study were conducted in R 4.3.0 with the packages “TwoSampleMR,” “MendelianRandomization” and “MRPRESSO.”

### Genetic correlation analysis

Finally, we performed LDSC analyses on the FDR-corrected positive results of univariate analyses to assess the genetic correlation (Rg) of the 2 phenotypes in each causal pair, using LDSC software package^1^ (19, 20). The statistically significant association is defined to be *p* < 0.05.

## Results

**Figure 2:**
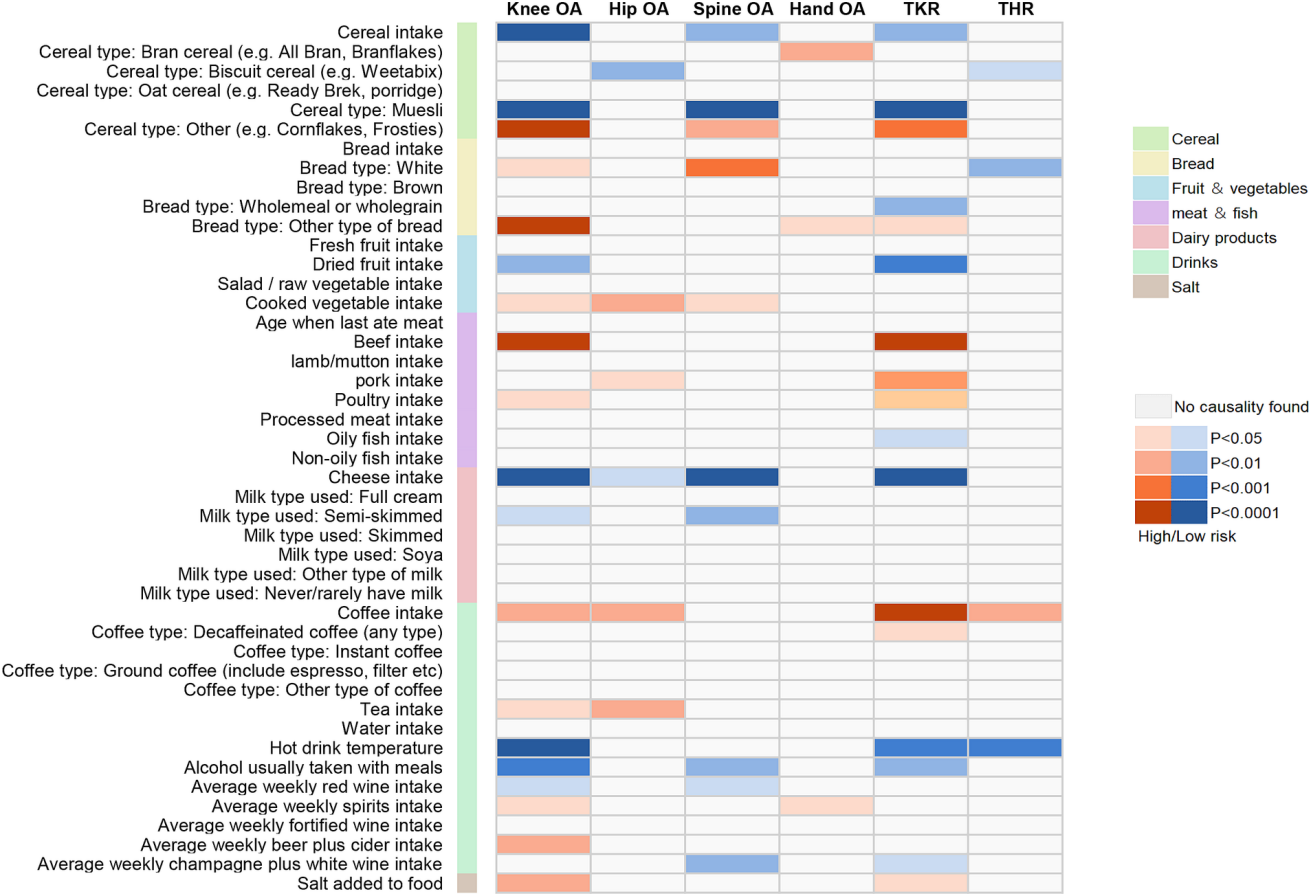
Mendelian randomization associations between the 45 dietary intakes and the 6 osteoarthritis phenotypes using IVW method.

**Figure 3:**
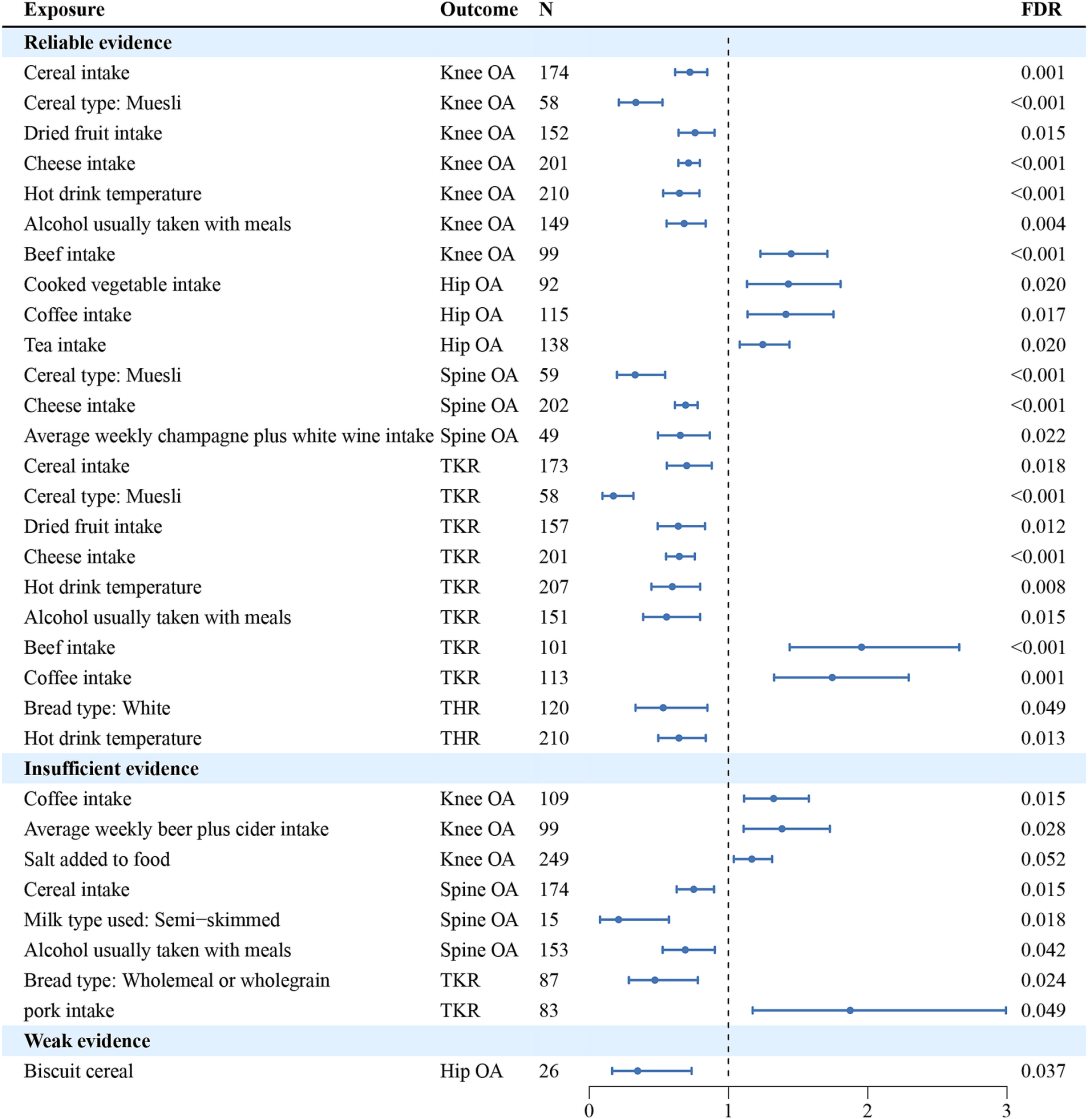
The assessment of the strength of potential causal associations after *p*-value correction and sensitivity analysis, showing the results using the IVW method. N denotes the number of SNPs used for each exposure.

In univariate analyses, we analyzed the causal association of 45 dietary intake habits with four different sites of osteoarthritis and two types of end-stage osteoarthritis. Out of the 270 results analyzed, we identified 59 potential pieces of evidence for causality without pleiotropy. The results of the causal associations between the 45 dietary intakes and the 6 osteoarthritis phenotypes using IVW method were presented in Figure 2; Supplementary Tables S2–S4, and the results of sensitivity analyses showed in Supplementary Tables S5–S10. Using the FDR method to correct the *p*-values, we identified 23 reliable causal evidence, 8 insufficient causal evidence, and 1 weak causal association, as determined by the results of sensitivity analyses. In reliable causal associations, 6 dietary intake habits were associated with the lower risk of Knee OA: cereal (OR = 0.724, 95%CI:0.617–0.848, *p* = 6.34E-05, FDR = 0.001), muesli (OR = 0.335, 95%CI:0.213–0.526, *p* = 2.12E-06, FDR < 0.001), dried fruit (OR = 0.760, 95%CI:0.642–0.900, *p* = 1.50E-03, FDR = 0.015) and cheese intake (OR = 0.713, 95%CI:0.641–0.794, *p* = 7.25E-10, FDR < 0.001), hot drink temperature (OR = 0.649, 95%CI:0.531–0.792, *p* = 2.06E-05, FDR < 0.001) and alcohol usually taken with meals (OR = 0.682, 95%CI:0.556–0.836, *p* = 2.40E-4, FDR = 0.004), while beef intake (OR = 1.450, 95%CI:1.230–1.711, *p* = 9.96E-06, FDR < 0.001) was positively associated with Knee OA. There were 3 dietary habits associated with Hip OA, all of which could increase the risk of Hip OA: cooked vegetable (OR = 1.431, 95%CI:1.134–1.805, *p* = 2.55E-03, FDR = 0.02), coffee (OR = 1.413, 95%CI:1.137–1.754, *p* = 1.79E-03, FDR = 0.017) and tea intake (OR = 1.247, 95%CI:1.081–1.438, *p* = 2.46E-03, FDR = 0.02). For Spine OA, 3 dietary intakes were associated with decreasing its risk: muesli (OR = 0.330, 95%CI:0.199–0.545, *p* = 1.53E-05, FDR < 0.001), cheese (OR = 0.692, 95%CI:0.616–0.779, *p* = 8.89E-10, FDR < 0.001) and average weekly champagne plus white wine intake (OR = 0.654, 95%CI:0.494–0.866, *p* = 3.02E-03, FDR = 0.022). There were 6 dietary intakes linked to the reduced risk for TKR: cereal (OR = 0.701, 95%CI:0.558–0.880, *p* = 2.24E-03, FDR = 0.018), muesli (OR = 0.174, 95%CI:0.095–0.318, *p* = 1.27E-08, FDR < 0.001), dried fruit (OR = 0.640, 95%CI:0.492–0.832, *p* = 8.58E-04, FDR = 0.012) and cheese intake (OR = 0.647, 95%CI:0.552–0.758, *p* = 7.69E-08, FDR < 0.001), hot drink temperature (OR = 0.597, 95%CI:0.447–0.797, *p* = 4.69E-04, FDR = 0.008) and alcohol usually taken with meals (OR = 0.556, 95%CI:0.388–0.796, *p* = 1.37E-03, FDR = 0.015), while beef (OR = 1.956, 95%CI:1.440–2.657, *p* = 1.78E-05, FDR < 0.001) and coffee intake (OR = 1.746, 95%CI:1.328–2.295, *p* = 6.50E-05, FDR = 0.001) were positively associated. Besides, white bread type intake (OR = 0.532, 95%CI:0.333–0.849, *p* = 8.19E-03, FDR = 0.049) and hot drink temperature (OR = 0.645, 95%CI:0.496–0.837, *p* = 9.91E-04, FDR = 0.013) were associated with a decrease in risk of THR. There were 8 insufficient evidence for Knee OA, Spine OA and TKR. Coffee intake (OR = 1.325, 95%CI:1.112–1.577, *p* = 1.60E-03, FDR = 0.015), average weekly beer plus cider intake (OR = 1.385, 95%CI:1.109–1.729, *p* = 4.03E-03, FDR = 0.028) and salt added to food (OR = 1.168, 95%CI:1.039–1.314, *p* = 9.56E-03, FDR = 0.052) were associated with a increase in risk of Knee OA, while cereal intake (OR = 0.751, 95%CI:0.628–0.897, *p* = 1.55E-03, FDR = 0.015), semi-skimmed milk type used (OR = 0.211, 95%CI:0.078–0.573, *p* = 2.24E-03, FDR = 0.018) and alcohol usually taken with meals (OR = 0.690, 95%CI:0.528–0.902, *p* = 6.73E-03, FDR = 0.042) were associated with a decrease in risk of Spine OA. For TKR, wholemeal or wholegrain bread type (OR = 0.472, 95%CI:0.285–0.78, *p* = 3.43E-03, FDR = 0.024) was associated with decreasing its risk, while pork intake (OR = 1.874, 95%CI: 1.174–2.993, *p* = 8.51E-03, FDR = 0.049) might increase the risk. And there was a weak evidence of negative associations between biscuit cereal intake and Hip OA (OR = 0.348, 95%CI: 0.165–0.735, *p* = 5.68E-03, FDR = 0.037). FDR results of associations between dietary habits and subtypes related to osteoarthritis showed in Supplementary Table S11. Figure 3 illustrates the assessment of the strength of potential causal associations after *p*-value correction and sensitivity analysis, showing the results using the IVW method.

**Figure 4:**
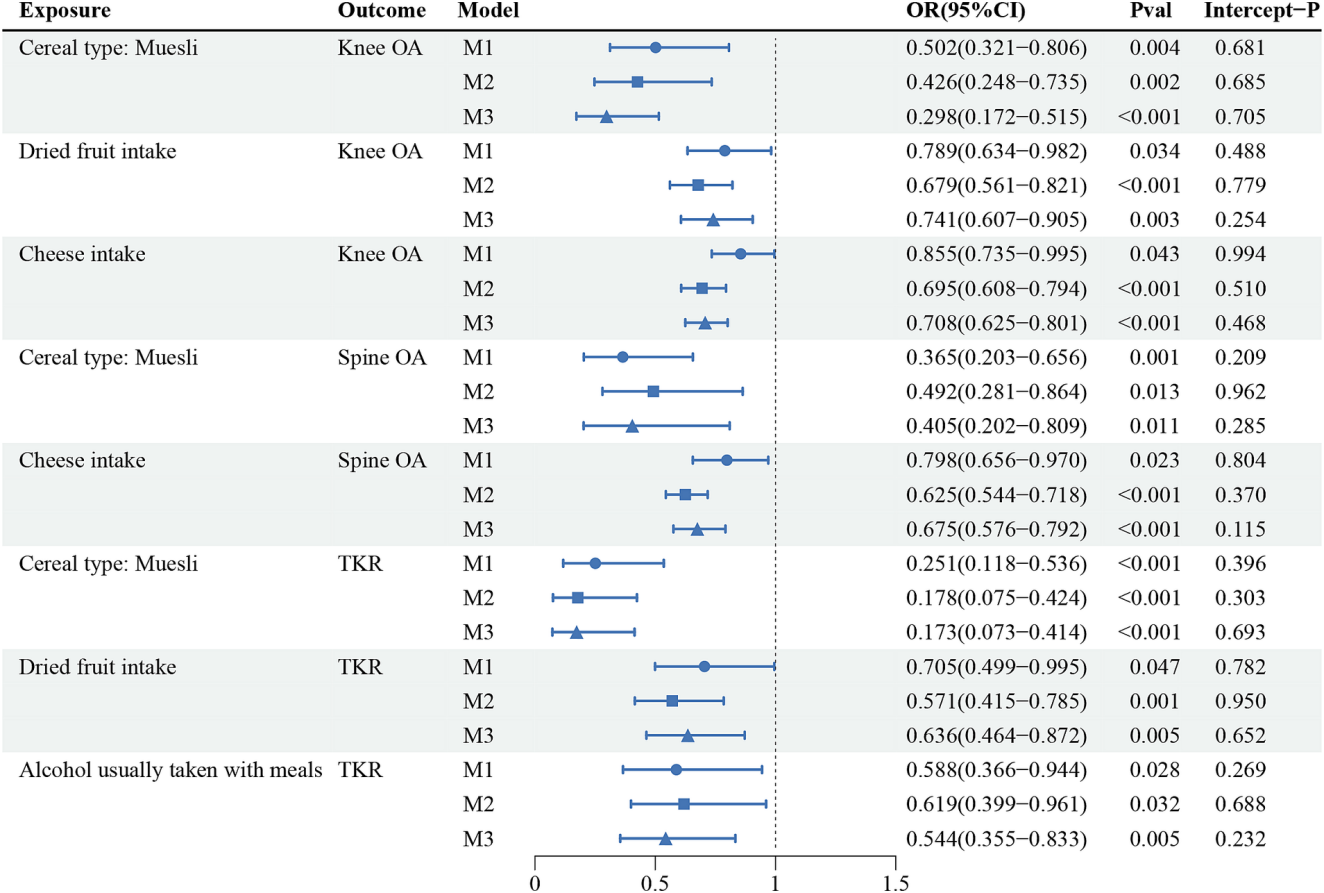
The causal relationships that still held after further multivariate analyses of 23 reliable evidence.

The multivariate analyses were further analyzed for the 23 reliable causal relationships screened. After adjusting separately for BMI, TB-BMD, and ever smoked, which were common risk factors for osteoarthritis, 8 causality estimates remained statistically significant: the effects of muesli intake on Knee OA, Spine OA and TKR; the effects of dried fruit intake on Knee OA and TKR; the effects of eating cheese on Knee OA and Spine OA; and the effect of alcohol usually taken with meals on TKR. Consistent with the results of univariate MR analyses, the multivariable MR analysis results supported that the 4 dietary intake habits above were protective against OA without pleiotropy in MR Egger intercept tests. The results of multivariable MR were shown in Figure 4; Supplementary Table S12.

Finally, we performed the LDSC analyses on all positive results corrected for FDR. The LDSC results showed significant genetic correlations for all causal relationships involving the Knee OA, Spine OA and TKR phenotypes, which genetically validated the potential for dietary management of osteoarthritis of the knee and osteoarthritis of the spine. And we also found that of the 8 causal relationships ultimately screened by multivariate analyses, all exposures were also significantly genetically correlated with their respective corresponding outcomes, as shown in Table 2. All results of LDSC analyses were shown in Supplementary Table S13.

**Table 2:** LDSC analysis results of 8 causal associations in Figure 4.

Phenotype 1	Phenotype 2	Rg	Se	Pval
Cereal type: Muesli	Knee OA	−0.277	0.037	4.48E-14
Dried fruit intake	Knee OA	−0.155	0.032	1.64E-06
Cheese intake	Knee OA	−0.208	0.029	9.67E-13
Cereal type: Muesli	Spine OA	−0.331	0.048	5.87E-12
Cheese intake	Spine OA	−0.327	0.046	7.73E-13
Cereal type: Muesli	TKR	−0.307	0.042	1.60E-13
Dried fruit intake	TKR	−0.212	0.037	7.79E-09
Alcohol usually taken with meals	TKR	−0.206	0.034	1.15E-09

Rg, genetic correlation.

## Discussion

### Principal findings

We analyzed the overall effect of 45 common dietary intake habits on 6 subtypes related to osteoarthritis and identified 59 potential causal associations, including 23 reliable associations, 8 causally insufficient associations, and 1 weak association. We found that cheese intake was negatively associated with the risk of most osteoarthritis phenotypes, whereas coffee intake was positively associated. From the osteoarthritis perspective, we found that the occurrence of Knee OA and TKR (end-stage Knee OA) was influenced by many dietary factors. Some dietary intakes were associated with risk of Spinal OA, while most dietary intake habits were not significantly associated with other osteoarthritis phenotypes. Using multivariate analyses, we identified that 4 dietary intake habits (muesli, dried fruit, cheese intake and alcohol usually taken with meals) were associated with a reduced risk of osteoarthritis and that there might be underlying mechanisms other than those influenced through BMI, TB-BMD and smoking status. And the results of LDSC showed significant genetic correlations in each of the 8 causal pairs.

### Comparisons with other studies

For the effect of coffee and tea intake on OA, our findings are consistent with previous similar univariate MR studies (10, 11). For alcohol intake, a previous MR study found the frequency of alcohol intake to be a risk factor for hip and knee osteoarthritis (12). The exposure of our study regarding alcohol intake was “alcohol usually taken with meals,” which was found to be a protective factor for KOA, TKR and THR. Subsequently when we categorized the varieties of alcohol in detail, we discovered that different varieties of alcohol had different effects on osteoarthritis: red wine and champagne plus white wine played a potentially protective role in osteoarthritis, whereas spirits and beer plus cider might play a potentially harmful role in osteoarthritis. The reason for the inconsistency with previous MR analyses regarding alcohol intake is that previous studies did not differentiate between types of alcohol and the outcome phenotype did not differentiate between hip and knee osteoarthritis. In contrast, our study differentiated osteoarthritis at each site in detail and differentiated dietary categories in as much detail as possible while ensuring that IVs were sufficient. We found no other MR studies exploring the causal relationship between dietary factors and osteoarthritis.

In previous observational analyses, higher whole grain intake was found to be related with a lower risk of knee osteoarthritis (6), which is consistent with our findings. In addition, a recent study covering 47 dairy products found a significant negative association between cheese and whole milk intake and Knee OA (7). This is consistent with the results of our analyses of the association between cheese intake and Knee OA, but we did not find a genetically predicted significant association between whole milk intake and Knee OA; instead, the potential effect of semi-skimmed milk on Knee OA and Spine OA was identified. For meat intake, a study found that the high intake of fresh red meat reduced the risk of THR (21), however, the similar association was not found in our study and we found that most of the meat intake might increase the risk of OA, with the positive association between beef intake on Knee OA and TKR being reliable evidence. It is possible that these differences in findings are the result of bias due to the inability of observational studies to completely avoid confounders.

### Potential mechanisms

In previous studies, grains have been shown to be a beneficial dietary factor in the management of chronic diseases, with positive effects on a number of cancers, cardiovascular diseases and overweight and obesity (22–24). Cereals provide the body with soluble fiber b-glucan, which not only reduces serum cholesterol, but also controls blood glucose levels and insulin response and thus indirectly controls BMI (25). In our study total cereal intake was significantly associated with Knee OA and TKR, and the results were found to be non-significant after adjusting for BMI, suggesting that BMI played an important mediating role in this association. However, the protective effect of muesli on Knee OA, Spine OA and TKR remained significant after controlling for the 3 risk factors, suggesting other possible mechanisms for the effect of grains on osteoarthritis. Muesli is rich in phenolic substances as well as flavonoids, which can exert antioxidant properties in the human body through mechanisms such as 2,2-diphenyl-1-trinitrohydrazine free radical scavenging and inhibition of iron chelating activity (26). A study examined total phenols, flavonoids, and antioxidant activity in cereal flakes and found that flakes and muesli made from Dickkopf wheat and red wheat showed the highest total phenolics and flavonoids content as well as antioxidant activity, whereas the lowest total phenolics and flavonoids content and antioxidant activity were measured in commercial flakes and muesli (27). The study might explain the results of our study in which there was a potential positive correlation between bran cereal and the risk of Hand OA.

Conventional views suggest that fresh fruits are beneficial for chronic diseases including osteoarthritis, however, our study did not find a significant association between fresh fruits and OA, but instead unexpectedly found that dried fruit intake was negatively associated with Knee OA and TKR risk. Dried fruits are obtained through a series of drying techniques on fresh fruits and their nutrient content remains similar to the equivalent fresh fruits but more concentrated (28, 29). From a nutritional point of view, a study comparing the nutrient content of dried and fresh fruits found that drying treatments concentrated polyphenol content and consequently antioxidant activity, and it was found that drying techniques might have differential concentration effects on different fruit varieties in dried fruits (30). For the effects of dried fruits on osteoarthritis, Basu et al. investigated the effects of dietary freeze-dried strawberries on obesity-related hormones, inflammatory biomarkers, and lipid peroxidation, and found that freeze-dried strawberries not only lowered serum TNF-α levels as well as some of the lipid peroxidation products in obese patients with knee OA (31), but also played a role in reducing knee pain. In addition, a large cross-sectional study based on NHANES showed that sultana consumers had higher whole grain intake, lower added sugar intake, and healthier overall diets (32), which may also be a potential factor in the negative association of dried fruit intake with the risk of developing osteoarthritis.

Cheese has long been favored as a healthy diet rich in protein as well as minerals. It has beneficial effects on the musculoskeletal system, and many studies have shown that daily dietary dairy intake significantly increases bone mineral content (33–35) and that cheese intake has a greater effect on increasing bone mass compared to taking calcium supplements (36). In addition to increasing bone mineral content, cheese is rich in whey protein, in which amino acid composition is very similar to that of skeletal muscle, which makes it an effective anabolic supplement for maintaining and building muscle mass (37). More and more studies are beginning to focus on the role of skeletal muscle in osteoarthritis, but it is not clear whether the effect of cheese intake on osteoarthritis is related to skeletal muscle (38, 39). In addition, cheese also produces bioactive compounds with antioxidant, antimicrobial, anti-inflammatory, and immunomodulatory properties during ripening and leads to changes in the intestinal flora after consumption, which may play a significant role as a preventive measure against osteoarthritis (40).

The effect of alcohol consumption on osteoarthritis in previous observational studies has been less consistent, probably mainly because of the plethora of confounding factors associated with alcohol consumption. It has been shown that excessive alcohol consumption can worsen osteoarthritis by increasing synovial fluid uric acid levels and elevating pro-inflammatory cytokine levels (41). On the contrary, several *in vitro* experiments have demonstrated that resveratrol in wine can have a protective effect on articular cartilage (42, 43) and polyphenols in alcoholic beverages have been shown to modulate the human intestinal flora and increase antioxidant activity (44), which supports the conclusions of our study. The association between alcohol consumption and osteoarthritis may require larger sample sizes and more detailed categorization in future studies. Our study also found a causal relationship between hot drink temperature and osteoarthritis, which was an interesting result. Hot drinking temperature is usually associated with metabolism, but to the best of our knowledge, the relationship between hot drinking temperature and osteoarthritis is not currently reported. A recent study provided experimental evidence of the effect of daily drinking temperature on cognitive function and the development of Alzheimer’s disease in mice (45). This study found that drinking water at 0°C reduces pepsin activity, leading to impaired function of the insulin signaling pathway, which may also be a potential metabolic mechanism by which the temperature of hot drinks affects osteoarthritis. In a rat model, it has been demonstrated that caffeine has a detrimental effect on chondrocytes by a mechanism that may be related to endogenous adenosine modulation and a reduction in circulating IGF-1 (46), which may be a potential mechanism by which coffee and tea promote the development of osteoarthritis. Furthermore, it has been shown that higher meat and total protein intake is associated with inflammatory polyarthritis (47). Although red meat is a rich source of protein, it is also rich in saturated fats and other unhealthy substances. It seems to be an accepted fact that excessive intake of red meat and salt may lead to a series of metabolic disorders in the body such as hypertension and hyperlipidaemia thereby increasing the risk of chronic diseases.

### Strength and limitations

To the best of our knowledge, this is the first study to investigate the causal relationship between multiple dietary intakes and osteoarthritis in various parts of the body by means of MR analysis, which can avoid confounding factors as well as reverse causality. The majority of previous studies on diet and osteoarthritis have focused on the impact of obesity on Knee OA due to dietary factors, with a small number of studies exploring the effect of a particular dietary intake on Knee OA. Our study took advantage of MR to include 45 dietary intakes as well as 6 osteoarthritis phenotypes, with exposure and outcome data from the largest sample size dataset available. And after controlling for recognized risk factors such as BMI, we found that certain dietary intakes were still associated with their counterparts in osteoarthritis, implying that there are more other potential mechanisms of dietary influence on osteoarthritis that need to be explored in future studies. In terms of dietary intake, we identified several new factors associated with osteoarthritis, such as dried fruit intake, hot drink temperature, and different drinking habits; and for osteoarthritis, we identified the potential for knee and spine osteoarthritis to be prevented, as well as managed, through diet. Our study provides evidence and references for future studies exploring the in-depth mechanisms of diet and osteoarthritis.

Our study also has some limitations. Firstly, due to lack of the age and gender information from the summary-level GWAS results, this study cannot conduct a stratified analysis of OA based on these factors. Secondly, dietary habits were assessed using a touch-screen questionnaire, which may lead to bias in the assessment as well as in the analyses. Thirdly, Causal associations with pleiotropy or inconsistent directions of the 4 MR analysis methods were not processed further, but rather excluded from the results by directly defining that no association was found, but this does not indicate that these dietary habits are absolutely unrelated to OA. Fourthly, dietary intake habits were used as the exposure phenotype and we did not examine the effect of nutrients on OA. However, a recent study emphasized the matrix effect produced by food (48). Food is complex and not just the sum of certain nutrients. Several studies have found that dairy products have a higher effect on skeletal bone mass than calcium supplements (49, 50), which also emphasizes the overall effect of food on health. Food can also influence health by modulating gut microbes. The gut-bone and gut-muscle axes have been demonstrated, and the gut microbes have an impact on bone beyond calcium and protein intake (51). In the future, we will give more attention to the diet-gut-bone/muscle axis to further explore in depth the potential mechanisms that exist. Fifthly, although our study provides statistical clues to the relationship between diet and OA, and a theoretical basis for subsequent experimental studies and mechanistic explorations, our results should be interpreted carefully. And a combination of evidence from multiple sources such as well-established trials and clinical findings is needed to confirm our findings in the future.

## Conclusion

Our study provides relatively solid evidence for a causal relationship between dietary factors and osteoarthritis. Most importantly, we identified that cereals, dried fruits, cheese and alcohol usually taken with meals intake remained negatively associated with some OA risk after controlling for BMI, TB-BMD and smoking status. In addition, we found the potential for multiple dietary interventions, management and prevention of knee and spine osteoarthritis. Our study has important implications for the prevention, management, and intervention of osteoarthritis in common sites through rational dietary modification.

## Data availability statement

The raw data supporting the conclusions of this article will be made available by the authors, without undue reservation.

## Author contributions

ZX: Data curation, Investigation, Methodology, Visualization, Writing – original draft. YQ: Supervision, Writing – review & editing.

## Footnotes

^1^https://github.com/bulik/ldsc
