# Supplementary material for: Is diet related to osteoarthritis? A univariable and multivariable Mendelian randomization study that investigates 45 dietary habits and osteoarthritis
Source: Front Nutr. 2023 Nov 16;10:1278079. doi: 10.3389/fnut.2023.1278079 (PMC10687195; doi:10.3389/fnut.2023.1278079)
Supplement: Supplementary file 1 [file Table_1.DOCX]

Supplementary Material

**Supplementary Table 1. Information of summary data of 45 dietary habits.**

| **Dietary habits** | **GWAS ID** | **Sample size** | **Types of diet** | **ACE touchscreen question** |
| --- | --- | --- | --- | --- |
| Cereal intake | ukb-b-15926 | 441,640 | Cereal | "How many bowls of cereal do you eat a WEEK?" |
| Cereal type: Bran cereal (e.g. All Bran, Branflakes) | ukb-d-1468_1 | 299,898 | Cereal | "What type of cereal do you mainly eat?"(If you eat more than one type of cereal, please select the one that you eat the most.) |
| Cereal type: Biscuit cereal (e.g. Weetabix) | ukb-d-1468_2 | 299,898 | Cereal |  |
| Cereal type: Oat cereal (e.g. Ready Brek, porridge) | ukb-d-1468_3 | 299,898 | Cereal |  |
| Cereal type: Muesli | ukb-d-1468_4 | 299,898 | Cereal |  |
| Cereal type: Other (e.g. Cornflakes, Frosties) | ukb-d-1468_5 | 299,898 | Cereal |  |
| Bread intake | ukb-b-11348 | 452,236 | Bread | "How many slices of bread do you eat each WEEK?" (For other types of bread: - one bread roll = 2 slices; - one pitta bread = 2 slices) |
| Bread type: White | ukb-d-1448_1 | 348,424 | Bread | "What type of bread do you mainly eat?"(If you eat more than one type of bread, please select the one that you eat the most.) |
| Bread type: Brown | ukb-d-1448_2 | 348,424 | Bread |  |
| Bread type: Wholemeal or wholegrain | ukb-d-1448_3 | 348,424 | Bread |  |
| Bread type: Other type of bread | ukb-d-1448_4 | 348,424 | Bread |  |
| Fresh fruit intake | ukb-b-3881 | 446,462 | Fruit and vegetables | "About how many pieces of FRESH fruit would you eat per DAY? (Count one apple, one banana, 10 grapes etc as one piece; put '0' if you do not eat any)" |
| Dried fruit intake | ukb-b-16576 | 421,764 | Fruit and vegetables | "About how many pieces of DRIED fruit would you eat per DAY? (Count one prune, one dried apricot, 10 raisins as one piece; put '0' if you do not eat any)" |
| Salad / raw vegetable intake | ukb-b-1996 | 435,435 | Fruit and vegetables | "On average how many heaped tablespoons of SALAD or RAW vegetables would you eat per DAY? (Include lettuce, tomato in sandwiches; put '0' if you do not eat any)" |
| Cooked vegetable intake | ukb-b-8089 | 448,651 | Fruit and vegetables | "On average how many heaped tablespoons of COOKED vegetables would you eat per DAY? (Do not include potatoes; put '0' if you do not eat any)" |
| Age when last ate meat | ukb-b-9791 | 17,236 | Meat and fish | "How old were you when you last ate any kind of meat? (Enter "0" if you have never eaten meat in your lifetime)" |
| Beef intake | ukb-b-2862 | 461,053 | Meat and fish | "How often do you eat beef? (Do not count processed meats)" |
| lamb/mutton intake | ukb-b-14179 | 460,006 | Meat and fish | "How often do you eat lamb/mutton? (Do not count processed meats)" |
| pork intake | ukb-b-5640 | 460,162 | Meat and fish | "How often do you eat pork? (Do not count processed meats such as bacon or ham)" |
| Poultry intake | ukb-b-8006 | 461,900 | Meat and fish | "How often do you eat chicken, turkey or other poultry? (Do not count processed meats)" |
| Processed meat intake | ukb-b-6324 | 461,981 | Meat and fish | "How often do you eat processed meats (such as bacon, ham, sausages, meat pies, kebabs, burgers, chicken nuggets)?" |
| Oily fish intake | ukb-b-2209 | 460,443 | Meat and fish | "How often do you eat oily fish? (e.g. sardines, salmon, mackerel, herring)" |
| Non-oily fish intake | ukb-b-17627 | 460,880 | Meat and fish | "How often do you eat other types of fish? (e.g. cod, tinned tuna, haddock)" |
| Cheese intake | ukb-b-1489 | 451,486 | Diary products | "How often do you eat cheese? (Include cheese in pizzas, quiches, cheese sauce etc)" |
| Milk type used: Full cream | ukb-d-1418_1 | 360,806 | Diary products | "What type of milk do you mainly use?"If you use more than one type of milk, please select the one that you drink the most. |
| Milk type used: Semi-skimmed | ukb-d-1418_2 | 360,806 | Diary products |  |
| Milk type used: Skimmed | ukb-d-1418_3 | 360,806 | Diary products |  |
| Milk type used: Soya | ukb-d-1418_4 | 360,806 | Diary products |  |
| Milk type used: Other type of milk | ukb-d-1418_5 | 360,806 | Diary products |  |
| Milk type used: Never/rarely have milk | ukb-d-1418_6 | 360,806 | Diary products |  |
| Coffee intake | ukb-b-5237 | 428,860 | Drinks | "How many cups of coffee do you drink each DAY? (Include decaffeinated coffee)" |
| Coffee type: Decaffeinated coffee (any type) | ukb-d-1508_1 | 283,449 | Drinks | "What type of coffee do you usually drink?"If you drink more than one type of coffee, please select the one that you drink the most. |
| Coffee type: Instant coffee | ukb-d-1508_2 | 283,449 | Drinks |  |
| Coffee type: Ground coffee (include espresso, filter etc) | ukb-d-1508_3 | 283,449 | Drinks |  |
| Coffee type: Other type of coffee | ukb-d-1508_4 | 283,449 | Drinks |  |
| Tea intake | ukb-b-6066 | 447,485 | Drinks | "How many cups of tea do you drink each DAY? (Include black and green tea)" |
| Water intake | ukb-b-14898 | 427,588 | Drinks | "How many glasses of water do you drink each DAY? " |
| Hot drink temperature | ukb-b-14203 | 457,873 | Drinks | "How do you like your hot drinks? (Such as coffee or tea)" |
| Alcohol usually taken with meals | ukb-b-16878 | 235,645 | Drinks | "When you drink alcohol is it usually with meals?" |
| Average weekly red wine intake | ukb-b-5239 | 327,026 | Drinks | "In an average WEEK, how many glasses of RED wine would you drink? (There are six glasses in an average bottle)" |
| Average weekly spirits intake | ukb-b-1707 | 326,565 | Drinks | "In an average WEEK, how many measures of spirits or liqueurs would you drink? (there are 25 standard measures in a normal sized bottle; spirits include drinks such as whisky, gin, rum, vodka, brandy)" |
| Average weekly fortified wine intake | ukb-b-1070 | 327,563 | Drinks | "In an average WEEK, how many glasses of fortified wine would you drink? (There are 12 glasses in an average bottle) (Fortified wines include drinks such as sherry, port, vermouth)" |
| Average weekly beer plus cider intake | ukb-b-5174 | 327,634 | Drinks | "In an average WEEK, how many pints of beer or cider would you drink? (Include bitter, lager, stout, ale, Guinness)" |
| Average weekly champagne plus white wine intake | ukb-b-5716 | 326,801 | Drinks | "In an average WEEK, how many glasses of WHITE wine or champagne would you drink? (There are six glasses in an average bottle)" |
| Salt added to food | ukb-b-8121 | 462,630 | Salt | "Do you add salt to your food? (Do not include salt used in cooking)" |

**Supplementary Table 2.** **Causality of genetically determined 45 dietary habits on Knee OA and Hip OA using IVW.**

| **Exposure** | **Knee OA** | | | | | | **Hip OA** | | | | | |
| --- | --- | --- | --- | --- | --- | --- | --- | --- | --- | --- | --- | --- |
|  | **N snps** | **Method** | **OR** | **95%CI** | **Pval** | **Q_pval** | **N snps** | **Method** | **OR** | **95%CI** | **Pval** | **Q_pval** |
| Cereal intake | 174 | MRE | 0.723504 | 0.617-0.848 | 6.34E-05 | <0.05 | 174 | MRE | 0.926393 | 0.774-1.108 | 4.03E-01 | <0.05 |
| Cereal type: Bran cereal (e.g. All Bran, Branflakes) | 13 | FE | 1.639140 | 0.697-3.854 | 2.57E-01 | 0.770 | 13 | FE | 2.369675 | 0.814-6.895 | 1.13E-01 | 0.518 |
| Cereal type: Biscuit cereal (e.g. Weetabix) | 26 | FE | 0.889725 | 0.494-1.602 | 6.97E-01 | 0.181 | 26 | FE | 0.348235 | 0.165-0.735 | 5.68E-03 | 0.108 |
| Cereal type: Oat cereal (e.g. Ready Brek, porridge) | 21 | FE | 1.083174 | 0.572-2.05 | 8.06E-01 | 0.192 | 22 | FE | 1.386144 | 0.638-3.011 | 4.09E-01 | 0.300 |
| Cereal type: Muesli | 58 | MRE | 0.335033 | 0.213-0.526 | 2.12E-06 | <0.05 | 58 | FE | 0.706310 | 0.449-1.112 | 1.33E-01 | 0.305 |
| Cereal type: Other (e.g. Cornflakes, Frosties) | 53 | MRE | 2.811660 | 1.736-4.555 | 2.67E-05 | <0.05 | 51 | FE | 1.285630 | 0.785-2.105 | 3.18E-01 | 0.108 |
| Bread intake | 117 | MRE | 0.991598 | 0.855-1.15 | 9.11E-01 | <0.05 | 119 | MRE | 0.874093 | 0.729-1.048 | 1.46E-01 | <0.05 |
| Bread type: White | 114 | MRE | 1.503336 | 1.023-2.209 | 3.79E-02 | <0.05 | 119 | MRE | 0.727071 | 0.486-1.088 | 1.21E-01 | <0.05 |
| Bread type: Brown | 19 | MRE | 2.249581 | 0.631-8.018 | 2.11E-01 | <0.05 | 19 | FE | 1.637578 | 0.507-5.29 | 4.10E-01 | 0.463 |
| Bread type: Wholemeal or wholegrain | 87 | MRE | 0.714176 | 0.515-0.991 | 4.38E-02 | <0.05 | 89 | MRE | 1.322374 | 0.853-2.05 | 2.12E-01 | <0.05 |
| Bread type: Other type of bread | 22 | FE | 14.600418 | 4.17-51.119 | 2.75E-05 | 0.126 | 22 | FE | 0.885337 | 0.181-4.335 | 8.81E-01 | 0.159 |
| Fresh fruit intake | 137 | MRE | 1.152123 | 0.91-1.459 | 2.39E-01 | <0.05 | 139 | MRE | 1.176019 | 0.865-1.598 | 3.00E-01 | <0.05 |
| Dried fruit intake | 152 | MRE | 0.760260 | 0.642-0.9 | 1.50E-03 | <0.05 | 155 | MRE | 0.974557 | 0.799-1.189 | 8.00E-01 | <0.05 |
| Salad / raw vegetable intake | 103 | MRE | 0.967808 | 0.757-1.237 | 7.94E-01 | <0.05 | 106 | MRE | 1.252601 | 0.95-1.651 | 1.10E-01 | <0.05 |
| Cooked vegetable intake | 93 | MRE | 1.247570 | 1.014-1.535 | 3.68E-02 | <0.05 | 92 | FE | 1.430699 | 1.134-1.805 | 2.55E-03 | 0.115 |
| Age when last ate meat | 11 | FE | 1.020092 | 0.927-1.122 | 6.83E-01 | 0.662 | 11 | FE | 0.991272 | 0.881-1.116 | 8.85E-01 | 0.155 |
| Beef intake | 99 | FE | 1.450459 | 1.23-1.711 | 9.96E-06 | 0.505 | 101 | MRE | 1.153620 | 0.868-1.533 | 3.24E-01 | <0.05 |
| lamb/mutton intake | 123 | MRE | 1.103392 | 0.891-1.367 | 3.68E-01 | <0.05 | 128 | MRE | 0.832135 | 0.645-1.073 | 1.57E-01 | <0.05 |
| pork intake | 81 | MRE | 1.513860 | 1.154-1.986 | 2.75E-03 | <0.05 | 81 | FE | 1.314960 | 1.013-1.708 | 3.99E-02 | 0.216 |
| Poultry intake | 81 | MRE | 1.312763 | 1.041-1.655 | 2.13E-02 | <0.05 | 80 | FE | 1.027497 | 0.823-1.282 | 8.10E-01 | 0.120 |
| Processed meat intake | 124 | MRE | 0.975303 | 0.823-1.156 | 7.73E-01 | <0.05 | 129 | MRE | 0.916320 | 0.771-1.089 | 3.22E-01 | <0.05 |
| Oily fish intake | 166 | MRE | 0.943205 | 0.821-1.084 | 4.09E-01 | <0.05 | 169 | MRE | 1.213355 | 1.05-1.402 | 8.87E-03 | <0.05 |
| Non-oily fish intake | 62 | MRE | 1.068137 | 0.795-1.435 | 6.62E-01 | <0.05 | 67 | MRE | 1.301357 | 0.956-1.772 | 9.45E-02 | <0.05 |
| Cheese intake | 201 | MRE | 0.713302 | 0.641-0.794 | 7.25E-10 | <0.05 | 201 | MRE | 0.874848 | 0.769-0.995 | 4.18E-02 | <0.05 |
| Milk type used: Full cream | 27 | FE | 0.869143 | 0.324-2.331 | 7.81E-01 | 0.260 | 24 | MRE | 1.326173 | 0.21-8.385 | 7.64E-01 | <0.05 |
| Milk type used: Semi-skimmed | 14 | FE | 0.384690 | 0.185-0.801 | 1.06E-02 | 0.078 | 13 | MRE | 1.158930 | 0.303-4.43 | 8.29E-01 | <0.05 |
| Milk type used: Skimmed | 26 | MRE | 2.137655 | 0.964-4.74 | 6.15E-02 | <0.05 | 28 | MRE | 0.758946 | 0.278-2.073 | 5.90E-01 | <0.05 |
| Milk type used: Soya | 23 | MRE | 0.162736 | 0.022-1.204 | 7.54E-02 | <0.05 | 25 | FE | 2.656618 | 0.462-15.27 | 2.74E-01 | 0.101 |
| Milk type used: Other type of milk | 13 | FE | 0.818373 | 0.029-22.9 | 9.06E-01 | 0.100 | 11 | FE | 18.355845 | 0.183-1.85E+03 | 2.16E-01 | 0.268 |
| Milk type used: Never/rarely have milk | 22 | FE | 1.360246 | 0.266-6.95 | 7.12E-01 | 0.307 | 22 | FE | 0.496330 | 0.066-3.755 | 4.97E-01 | 0.467 |
| Coffee intake | 109 | MRE | 1.324598 | 1.112-1.577 | 1.60E-03 | <0.05 | 115 | MRE | 1.412511 | 1.137-1.754 | 1.79E-03 | <0.05 |
| Coffee type: Decaffeinated coffee | 17 | FE | 1.658811 | 0.848-3.245 | 1.39E-01 | 0.396 | 17 | FE | 0.783142 | 0.334-1.836 | 5.74E-01 | 0.558 |
| Coffee type: Instant coffee | 29 | FE | 0.887938 | 0.582-1.354 | 5.81E-01 | 0.165 | 29 | FE | 0.700174 | 0.416-1.178 | 1.79E-01 | 0.463 |
| Coffee type: Ground coffee (include espresso, filter etc) | 99 | MRE | 0.576462 | 0.406-0.818 | 2.07E-03 | <0.05 | 99 | MRE | 0.908385 | 0.626-1.318 | 6.13E-01 | <0.05 |
| Coffee type: Other type of coffee | 6 | FE | 3.462932 | 0.087-137.303 | 5.08E-01 | 0.448 | 6 | FE | 23.672283 | 0.214-2.61E+03 | 1.87E-01 | 0.729 |
| Tea intake | 137 | MRE | 1.134153 | 1.001-1.285 | 4.82E-02 | <0.05 | 138 | MRE | 1.246731 | 1.081-1.438 | 2.46E-03 | <0.05 |
| Water intake | 156 | MRE | 1.146488 | 0.995-1.321 | 5.92E-02 | <0.05 | 158 | MRE | 1.178023 | 0.974-1.425 | 9.16E-02 | <0.05 |
| Hot drink temperature | 210 | MRE | 0.648644 | 0.531-0.792 | 2.06E-05 | <0.05 | 211 | MRE | 0.688563 | 0.557-0.852 | 5.81E-04 | <0.05 |
| Alcohol usually taken with meals | 149 | MRE | 0.681887 | 0.556-0.836 | 2.40E-04 | <0.05 | 150 | MRE | 0.800512 | 0.624-1.027 | 7.97E-02 | <0.05 |
| Average weekly red wine intake | 94 | MRE | 0.810932 | 0.682-0.965 | 1.80E-02 | <0.05 | 97 | MRE | 1.140729 | 0.935-1.392 | 1.95E-01 | <0.05 |
| Average weekly spirits intake | 43 | FE | 1.281100 | 1.053-1.558 | 1.32E-02 | 0.764 | 43 | FE | 1.267970 | 0.988-1.627 | 6.18E-02 | 0.058 |
| Average weekly fortified wine intake | 22 | FE | 1.059443 | 0.624-1.798 | 8.31E-01 | 0.321 | 21 | FE | 0.873337 | 0.439-1.736 | 6.99E-01 | 0.325 |
| Average weekly beer plus cider intake | 99 | MRE | 1.384664 | 1.109-1.729 | 4.03E-03 | <0.05 | 100 | MRE | 0.925811 | 0.729-1.175 | 5.26E-01 | <0.05 |
| Average weekly champagne plus white wine intake | 49 | FE | 0.844076 | 0.694-1.027 | 9.05E-02 | 0.057 | 48 | MRE | 0.728005 | 0.536-0.988 | 4.15E-02 | <0.05 |
| Salt added to food | 249 | MRE | 1.168079 | 1.039-1.314 | 9.56E-03 | <0.05 | 248 | MRE | 1.006523 | 0.891-1.137 | 9.17E-01 | <0.05 |

**Supplementary Table 3. Causality of genetically determined 45 dietary habits on Spine OA and Hand OA using IVW.**

| **Exposure** | **Spine OA** | | | | | | **Hand OA** | | | | | |
| --- | --- | --- | --- | --- | --- | --- | --- | --- | --- | --- | --- | --- |
|  | **N snps** | **Method** | **OR** | **95%CI** | **Pval** | **Q_pval** | **N snps** | **Method** | **OR** | **95%CI** | **Pval** | **Q_pval** |
| Cereal intake | 174 | MRE | 0.750576 | 0.628-0.897 | 1.55E-03 | <0.05 | 176 | MRE | 0.859357 | 0.686-1.076 | 1.86E-01 | <0.05 |
| Cereal type: Bran cereal (e.g. All Bran, Branflakes) | 13 | FE | 3.176696 | 0.946-10.663 | 6.14E-02 | 0.354 | 12 | FE | 12.964046 | 2.722-61.745 | 1.29E-03 | 0.767 |
| Cereal type: Biscuit cereal (e.g. Weetabix) | 27 | FE | 0.677631 | 0.296-1.55 | 3.57E-01 | 0.346 | 27 | FE | 1.220371 | 0.454-3.277 | 6.93E-01 | 0.765 |
| Cereal type: Oat cereal (e.g. Ready Brek, porridge) | 23 | FE | 1.492496 | 0.654-3.408 | 3.42E-01 | 0.233 | 23 | FE | 3.478929 | 1.249-9.69 | 1.71E-02 | 0.099 |
| Cereal type: Muesli | 59 | FE | 0.329785 | 0.199-0.545 | 1.53E-05 | 0.099 | 59 | FE | 0.714195 | 0.393-1.296 | 2.68E-01 | 0.319 |
| Cereal type: Other (e.g. Cornflakes, Frosties) | 55 | FE | 2.169484 | 1.266-3.717 | 4.82E-03 | 0.069 | 54 | MRE | 1.421328 | 0.624-3.235 | 4.02E-01 | <0.05 |
| Bread intake | 117 | FE | 1.031404 | 0.866-1.229 | 7.29E-01 | 0.052 | 119 | FE | 1.095913 | 0.888-1.352 | 3.93E-01 | 0.184 |
| Bread type: White | 122 | MRE | 2.142507 | 1.45-3.165 | 1.29E-04 | <0.05 | 121 | MRE | 0.903325 | 0.536-1.523 | 7.03E-01 | <0.05 |
| Bread type: Brown | 20 | FE | 0.583995 | 0.164-2.082 | 4.07E-01 | 0.591 | 20 | FE | 1.519376 | 0.327-7.05 | 5.93E-01 | 0.430 |
| Bread type: Wholemeal or wholegrain | 92 | MRE | 0.777848 | 0.507-1.193 | 2.50E-01 | <0.05 | 93 | MRE | 0.928435 | 0.552-1.56 | 7.79E-01 | <0.05 |
| Bread type: Other type of bread | 22 | FE | 4.861499 | 0.844-27.99 | 7.66E-02 | 0.668 | 22 | FE | 13.272395 | 1.433-122.945 | 2.28E-02 | 0.528 |
| Fresh fruit intake | 141 | MRE | 0.807934 | 0.603-1.082 | 1.53E-01 | <0.05 | 139 | MRE | 0.987379 | 0.705-1.383 | 9.41E-01 | <0.05 |
| Dried fruit intake | 156 | MRE | 0.726960 | 0.594-0.889 | 1.94E-03 | <0.05 | 157 | MRE | 0.892243 | 0.699-1.139 | 3.61E-01 | <0.05 |
| Salad / raw vegetable intake | 108 | FE | 0.933463 | 0.719-1.211 | 6.04E-01 | 0.058 | 109 | MRE | 1.133391 | 0.787-1.631 | 5.00E-01 | <0.05 |
| Cooked vegetable intake | 94 | FE | 1.320251 | 1.021-1.707 | 3.39E-02 | 0.067 | 95 | FE | 1.553448 | 1.138-2.12 | 5.52E-03 | 0.850 |
| Age when last ate meat | 11 | FE | 1.014584 | 0.886-1.161 | 8.34E-01 | 0.764 | 11 | FE | 1.060463 | 0.901-1.248 | 4.79E-01 | 0.309 |
| Beef intake | 102 | FE | 1.148259 | 0.914-1.442 | 2.35E-01 | 0.189 | 101 | FE | 0.759459 | 0.576-1.001 | 5.12E-02 | 0.119 |
| lamb/mutton intake | 130 | MRE | 0.837794 | 0.641-1.095 | 1.95E-01 | <0.05 | 129 | MRE | 0.942257 | 0.69-1.287 | 7.09E-01 | <0.05 |
| pork intake | 84 | MRE | 1.363174 | 0.986-1.885 | 6.09E-02 | <0.05 | 84 | MRE | 1.038615 | 0.689-1.566 | 8.56E-01 | <0.05 |
| Poultry intake | 79 | MRE | 1.376024 | 1.014-1.866 | 4.01E-02 | <0.05 | 81 | FE | 1.169008 | 0.865-1.579 | 3.09E-01 | 0.060 |
| Processed meat intake | 129 | MRE | 0.946140 | 0.776-1.154 | 5.85E-01 | <0.05 | 129 | MRE | 0.871480 | 0.686-1.107 | 2.59E-01 | <0.05 |
| Oily fish intake | 170 | FE | 1.019290 | 0.884-1.175 | 7.92E-01 | 0.098 | 169 | FE | 1.098017 | 0.923-1.306 | 2.91E-01 | 0.597 |
| Non-oily fish intake | 66 | FE | 0.931029 | 0.699-1.241 | 6.26E-01 | 0.053 | 68 | FE | 0.896092 | 0.635-1.265 | 5.33E-01 | 0.076 |
| Cheese intake | 202 | FE | 0.692318 | 0.616-0.779 | 8.89E-10 | 0.206 | 203 | FE | 0.945679 | 0.821-1.089 | 4.37E-01 | 0.074 |
| Milk type used: Full cream | 29 | FE | 1.456266 | 0.392-5.412 | 5.75E-01 | 0.543 | 28 | FE | 0.533482 | 0.105-2.712 | 4.49E-01 | 0.249 |
| Milk type used: Semi-skimmed | 15 | FE | 0.211386 | 0.078-0.573 | 2.24E-03 | 0.064 | 15 | FE | 1.164390 | 0.349-3.889 | 8.05E-01 | 0.550 |
| Milk type used: Skimmed | 30 | FE | 1.314207 | 0.617-2.801 | 4.79E-01 | 0.267 | 28 | FE | 0.816587 | 0.316-2.113 | 6.76E-01 | 0.372 |
| Milk type used: Soya | 25 | FE | 0.710661 | 0.102-4.967 | 7.31E-01 | 0.793 | 25 | FE | 0.687127 | 0.067-7.072 | 7.52E-01 | 0.708 |
| Milk type used: Other type of milk | 13 | FE | 10.045798 | 0.097-1.04E+03 | 3.30E-01 | 0.939 | 12 | FE | 0.458459 | 0.001-201.312 | 8.02E-01 | 0.156 |
| Milk type used: Never/rarely have milk | 21 | FE | 0.406221 | 0.04-4.098 | 4.45E-01 | 0.100 | 22 | FE | 0.660510 | 0.041-10.688 | 7.70E-01 | 0.797 |
| Coffee intake | 116 | MRE | 1.153955 | 0.924-1.441 | 2.07E-01 | <0.05 | 116 | FE | 1.436304 | 1.154-1.787 | 1.17E-03 | 0.086 |
| Coffee type: Decaffeinated coffee (any type) | 18 | FE | 1.816580 | 0.746-4.423 | 1.89E-01 | 0.151 | 18 | FE | 1.497043 | 0.504-4.448 | 4.68E-01 | 0.492 |
| Coffee type: Instant coffee | 34 | FE | 0.721317 | 0.425-1.224 | 2.26E-01 | 0.075 | 33 | MRE | 0.548752 | 0.24-1.255 | 1.55E-01 | <0.05 |
| Coffee type: Ground coffee (include espresso, filter etc) | 105 | MRE | 0.574502 | 0.38-0.868 | 8.43E-03 | <0.05 | 103 | MRE | 0.992587 | 0.619-1.593 | 9.75E-01 | <0.05 |
| Coffee type: Other type of coffee | 6 | FE | 0.325452 | 0.002-52.03 | 6.65E-01 | 0.270 | 6 | FE | 6.157376 | 0.01-3.94E+03 | 5.81E-01 | 0.926 |
| Tea intake | 140 | MRE | 1.124599 | 0.963-1.313 | 1.37E-01 | <0.05 | 141 | MRE | 1.200459 | 1.001-1.439 | 4.84E-02 | <0.05 |
| Water intake | 161 | MRE | 1.065960 | 0.883-1.288 | 5.07E-01 | <0.05 | 160 | MRE | 1.028184 | 0.817-1.295 | 8.13E-01 | <0.05 |
| Hot drink temperature | 213 | MRE | 0.684529 | 0.541-0.866 | 1.57E-03 | <0.05 | 208 | MRE | 0.878435 | 0.66-1.17 | 3.75E-01 | <0.05 |
| Alcohol usually taken with meals | 153 | MRE | 0.689981 | 0.528-0.902 | 6.73E-03 | <0.05 | 153 | MRE | 0.870958 | 0.626-1.213 | 4.13E-01 | <0.05 |
| Average weekly red wine intake | 98 | MRE | 0.799077 | 0.642-0.995 | 4.47E-02 | <0.05 | 98 | MRE | 1.057263 | 0.812-1.377 | 6.79E-01 | <0.05 |
| Average weekly spirits intake | 42 | FE | 1.309666 | 0.989-1.734 | 5.95E-02 | 0.055 | 44 | FE | 1.488833 | 1.073-2.067 | 1.74E-02 | 0.390 |
| Average weekly fortified wine intake | 22 | MRE | 1.080047 | 0.416-2.801 | 8.74E-01 | <0.05 | 22 | FE | 1.097012 | 0.441-2.726 | 8.42E-01 | 0.337 |
| Average weekly beer plus cider intake | 101 | MRE | 1.042670 | 0.8-1.36 | 7.58E-01 | <0.05 | 100 | FE | 0.979747 | 0.746-1.287 | 8.83E-01 | 0.398 |
| Average weekly champagne plus white wine intake | 49 | FE | 0.654135 | 0.494-0.866 | 3.02E-03 | 0.520 | 49 | FE | 0.894157 | 0.639-1.252 | 5.15E-01 | 0.532 |
| Salt added to food | 250 | MRE | 1.161209 | 0.996-1.353 | 5.57E-02 | <0.05 | 251 | MRE | 1.076477 | 0.912-1.271 | 3.84E-01 | <0.05 |

**Supplementary Table 4. Causality of genetically determined 45 dietary habits on TKR and THR using IVW.**

| **Exposure** | **TKR** | | | | | | **THR** | | | | | |
| --- | --- | --- | --- | --- | --- | --- | --- | --- | --- | --- | --- | --- |
|  | **N snps** | **Method** | **OR** | **95%CI** | **Pval** | **Q_pval** | **N snps** | **Method** | **OR** | **95%CI** | **Pval** | **Q_pval** |
| Cereal intake | 173 | MRE | 0.700634 | 0.558-0.88 | 2.24E-03 | <0.05 | 173 | MRE | 1.067780 | 0.873-1.305 | 5.22E-01 | <0.05 |
| Cereal type: Bran cereal (e.g. All Bran, Branflakes) | 13 | FE | 2.379185 | 0.555-10.202 | 2.43E-01 | 0.424 | 13 | FE | 1.649502 | 0.447-6.091 | 4.53E-01 | 0.955 |
| Cereal type: Biscuit cereal (e.g. Weetabix) | 26 | FE | 0.634134 | 0.24-1.676 | 3.58E-01 | 0.241 | 26 | FE | 0.369833 | 0.152-0.899 | 2.81E-02 | 0.059 |
| Cereal type: Oat cereal (e.g. Ready Brek, porridge) | 22 | FE | 1.133349 | 0.415-3.093 | 8.07E-01 | 0.350 | 22 | FE | 0.688507 | 0.276-1.716 | 4.23E-01 | 0.084 |
| Cereal type: Muesli | 58 | FE | 0.173943 | 0.095-0.318 | 1.27E-08 | 0.175 | 58 | FE | 0.801358 | 0.464-1.383 | 4.26E-01 | 0.166 |
| Cereal type: Other (e.g. Cornflakes, Frosties) | 53 | MRE | 3.984555 | 1.818-8.731 | 5.52E-04 | <0.05 | 53 | FE | 1.527790 | 0.855-2.729 | 1.52E-01 | 0.117 |
| Bread intake | 118 | MRE | 1.113465 | 0.863-1.437 | 4.09E-01 | <0.05 | 118 | MRE | 0.882431 | 0.708-1.099 | 2.65E-01 | <0.05 |
| Bread type: White | 117 | MRE | 1.630061 | 0.951-2.793 | 7.53E-02 | <0.05 | 120 | MRE | 0.531479 | 0.333-0.849 | 8.19E-03 | <0.05 |
| Bread type: Brown | 20 | FE | 2.583634 | 0.581-11.485 | 2.12E-01 | 0.240 | 19 | FE | 0.748940 | 0.187-3.006 | 6.83E-01 | 0.670 |
| Bread type: Wholemeal or wholegrain | 87 | MRE | 0.471752 | 0.285-0.78 | 3.43E-03 | <0.05 | 89 | MRE | 1.918670 | 1.108-3.322 | 2.00E-02 | <0.05 |
| Bread type: Other type of bread | 22 | FE | 9.415116 | 1.219-72.713 | 3.16E-02 | 0.150 | 22 | FE | 0.461075 | 0.072-2.965 | 4.15E-01 | 0.055 |
| Fresh fruit intake | 139 | MRE | 1.192775 | 0.849-1.676 | 3.10E-01 | <0.05 | 140 | MRE | 1.173293 | 0.84-1.639 | 3.48E-01 | <0.05 |
| Dried fruit intake | 157 | MRE | 0.639663 | 0.492-0.832 | 8.58E-04 | <0.05 | 157 | MRE | 1.112648 | 0.891-1.39 | 3.47E-01 | <0.05 |
| Salad / raw vegetable intake | 106 | MRE | 0.903325 | 0.634-1.287 | 5.74E-01 | <0.05 | 106 | MRE | 1.160675 | 0.828-1.627 | 3.87E-01 | <0.05 |
| Cooked vegetable intake | 94 | MRE | 1.233489 | 0.831-1.831 | 2.98E-01 | <0.05 | 94 | FE | 1.251895 | 0.947-1.655 | 1.14E-01 | 0.109 |
| Age when last ate meat | 11 | FE | 0.949882 | 0.813-1.11 | 5.17E-01 | 0.460 | 11 | FE | 0.998304 | 0.868-1.148 | 9.81E-01 | 0.126 |
| Beef intake | 101 | MRE | 1.955990 | 1.44-2.657 | 1.78E-05 | <0.05 | 100 | MRE | 1.302473 | 0.929-1.826 | 1.25E-01 | <0.05 |
| lamb/mutton intake | 127 | MRE | 1.386743 | 0.99-1.942 | 5.70E-02 | <0.05 | 127 | MRE | 0.806976 | 0.608-1.072 | 1.39E-01 | <0.05 |
| pork intake | 83 | MRE | 1.874366 | 1.174-2.993 | 8.51E-03 | <0.05 | 83 | FE | 1.254247 | 0.918-1.714 | 1.55E-01 | 0.068 |
| Poultry intake | 81 | MRE | 1.548059 | 1.09-2.199 | 1.47E-02 | <0.05 | 81 | MRE | 0.881072 | 0.648-1.198 | 4.19E-01 | <0.05 |
| Processed meat intake | 128 | MRE | 1.118835 | 0.883-1.417 | 3.52E-01 | <0.05 | 127 | MRE | 0.980978 | 0.802-1.199 | 8.51E-01 | <0.05 |
| Oily fish intake | 168 | MRE | 0.813198 | 0.662-0.999 | 4.89E-02 | <0.05 | 169 | MRE | 1.262517 | 1.042-1.53 | 1.74E-02 | <0.05 |
| Non-oily fish intake | 66 | FE | 1.096107 | 0.773-1.553 | 6.06E-01 | 0.075 | 66 | FE | 1.425854 | 1.04-1.956 | 2.77E-02 | 0.086 |
| Cheese intake | 201 | MRE | 0.647120 | 0.552-0.758 | 7.69E-08 | <0.05 | 201 | MRE | 0.952948 | 0.814-1.115 | 5.48E-01 | <0.05 |
| Milk type used: Full cream | 27 | FE | 1.632784 | 0.314-8.477 | 5.60E-01 | 0.148 | 25 | MRE | 0.938150 | 0.117-7.498 | 9.52E-01 | <0.05 |
| Milk type used: Semi-skimmed | 15 | FE | 0.718812 | 0.224-2.307 | 5.79E-01 | 0.067 | 14 | FE | 2.245356 | 0.75-6.723 | 1.48E-01 | 0.129 |
| Milk type used: Skimmed | 28 | FE | 1.054914 | 0.401-2.773 | 9.14E-01 | 0.309 | 27 | MRE | 0.554145 | 0.167-1.833 | 3.34E-01 | <0.05 |
| Milk type used: Soya | 25 | FE | 0.117620 | 0.012-1.173 | 6.81E-02 | 0.349 | 25 | FE | 4.279075 | 0.55-33.293 | 1.65E-01 | 0.079 |
| Milk type used: Other type of milk | 13 | FE | 0.030877 | 0-8.615 | 2.26E-01 | 0.614 | 12 | MRE | 152.920993 | 0.091-2.56E+05 | 1.84E-01 | <0.05 |
| Milk type used: Never/rarely have milk | 22 | MRE | 0.294958 | 0.007-12.072 | 5.19E-01 | <0.05 | 22 | FE | 0.807616 | 0.071-9.181 | 8.63E-01 | 0.082 |
| Coffee intake | 113 | MRE | 1.745634 | 1.328-2.295 | 6.50E-05 | <0.05 | 113 | MRE | 1.374868 | 1.081-1.749 | 9.58E-03 | <0.05 |
| Coffee type: Decaffeinated coffee (any type) | 18 | FE | 3.935695 | 1.355-11.431 | 1.18E-02 | 0.200 | 17 | FE | 0.617734 | 0.222-1.719 | 3.56E-01 | 0.674 |
| Coffee type: Instant coffee | 29 | FE | 0.897612 | 0.456-1.767 | 7.55E-01 | 0.193 | 29 | FE | 0.579984 | 0.312-1.079 | 8.56E-02 | 0.472 |
| Coffee type: Ground coffee (include espresso, filter etc) | 104 | MRE | 0.374005 | 0.213-0.656 | 5.99E-04 | <0.05 | 100 | MRE | 1.131496 | 0.729-1.757 | 5.82E-01 | <0.05 |
| Coffee type: Other type of coffee | 6 | FE | 0.185953 | 0-92.982 | 5.96E-01 | 0.907 | 6 | FE | 10.835030 | 0.039-2.98E+03 | 4.06E-01 | 0.570 |
| Tea intake | 139 | MRE | 1.278476 | 1.053-1.552 | 1.30E-02 | <0.05 | 139 | MRE | 1.133402 | 0.956-1.344 | 1.50E-01 | <0.05 |
| Water intake | 157 | FE | 1.228701 | 1.01-1.495 | 3.94E-02 | 0.068 | 159 | MRE | 1.344597 | 1.082-1.672 | 7.67E-03 | <0.05 |
| Hot drink temperature | 207 | MRE | 0.596940 | 0.447-0.797 | 4.69E-04 | <0.05 | 210 | MRE | 0.644644 | 0.496-0.837 | 9.91E-04 | <0.05 |
| Alcohol usually taken with meals | 151 | MRE | 0.555796 | 0.388-0.796 | 1.38E-03 | <0.05 | 150 | MRE | 0.942512 | 0.677-1.313 | 7.26E-01 | <0.05 |
| Average weekly red wine intake | 96 | MRE | 0.834848 | 0.64-1.088 | 1.82E-01 | <0.05 | 97 | MRE | 1.267239 | 0.999-1.607 | 5.07E-02 | <0.05 |
| Average weekly spirits intake | 43 | FE | 1.127264 | 0.809-1.571 | 4.79E-01 | 0.482 | 42 | FE | 1.133597 | 0.841-1.528 | 4.11E-01 | 0.097 |
| Average weekly fortified wine intake | 22 | FE | 1.376886 | 0.557-3.406 | 4.89E-01 | 0.644 | 21 | FE | 0.706447 | 0.306-1.629 | 4.15E-01 | 0.051 |
| Average weekly beer plus cider intake | 99 | MRE | 1.147353 | 0.831-1.583 | 4.03E-01 | <0.05 | 99 | MRE | 0.948617 | 0.711-1.265 | 7.19E-01 | <0.05 |
| Average weekly champagne plus white wine intake | 49 | FE | 0.709080 | 0.508-0.989 | 4.31E-02 | 0.053 | 48 | MRE | 0.652535 | 0.454-0.938 | 2.10E-02 | <0.05 |
| Salt added to food | 248 | MRE | 1.200473 | 1.001-1.44 | 4.86E-02 | <0.05 | 250 | MRE | 1.001538 | 0.868-1.155 | 9.83E-01 | <0.05 |

**Supplementary Table 5. Causality of genetically determined 45 dietary habits on Knee OA in sensitivity analysis.**

| **Exposure** | **N snps** | **Weighted median** | | | **MR egger** | | | | | **MR Presso** | | | |
| --- | --- | --- | --- | --- | --- | --- | --- | --- | --- | --- | --- | --- | --- |
|  |  | **OR** | **95%CI** | **Pval** | **OR** | **95%CI** | **Pval** | **Intercept** | **Intercept**  **Pval** | **OR** | **Pval** | **Distortion test** | **N outliers** |
| Cereal intake | 174 | 0.756181 | 0.632-0.905 | 2.30E-03 | 0.693571 | 0.391-1.231 | 2.13E-01 | 5.01E-04 | 0.881 | 0.752336 | 2.59E-04 | 0.965 | 2 |
| Cereal type: Bran cereal (e.g. All Bran, Branflakes) | 13 | 1.723764 | 0.534-5.567 | 3.63E-01 | 1.574739 | 0.231-10.724 | 6.52E-01 | 2.94E-04 | 0.964 | 1.438046 | 2.68E-01 | NA | 0 |
| Cereal type: Biscuit cereal (e.g. Weetabix) | 26 | 1.409506 | 0.587-3.386 | 4.43E-01 | 0.889253 | 0.163-4.843 | 8.93E-01 | 3.96E-06 | 0.999 | 0.919780 | 7.98E-01 | 0.201 | 1 |
| Cereal type: Oat cereal (e.g. Ready Brek, porridge) | 21 | 1.086852 | 0.441-2.68 | 8.56E-01 | 1.386740 | 0.262-7.345 | 7.05E-01 | -2.15E-03 | 0.750 | 1.221231 | 5.97E-01 | 0.217 | 1 |
| Cereal type: Muesli | 58 | 0.342072 | 0.195-0.599 | 1.78E-04 | 0.584097 | 0.137-2.49 | 4.70E-01 | -3.74E-03 | 0.432 | 0.341152 | 8.73E-06 | NA | 0 |
| Cereal type: Other (e.g. Cornflakes, Frosties) | 53 | 2.050774 | 1.123-3.747 | 1.95E-02 | 1.735733 | 0.369-8.176 | 4.89E-01 | 3.35E-03 | 0.524 | 2.811660 | 1.05E-04 | 0.487 | 1 |
| Bread intake | 117 | 0.903843 | 0.747-1.094 | 3.00E-01 | 0.758282 | 0.457-1.258 | 2.86E-01 | 3.69E-03 | 0.280 | 0.996044 | 9.57E-01 | 0.038 | 2 |
| Bread type: White | 114 | 1.252311 | 0.833-1.882 | 2.79E-01 | 1.738576 | 0.36-8.389 | 4.92E-01 | -9.76E-04 | 0.852 | 1.500906 | 3.37E-02 | 0.985 | 2 |
| Bread type: Brown | 19 | 0.821335 | 0.193-3.494 | 7.90E-01 | 0.087602 | 0.004-1.88 | 1.38E-01 | 1.83E-02 | 0.039 | 2.249581 | 2.27E-01 | NA | 0 |
| Bread type: Wholemeal or wholegrain | 87 | 0.748942 | 0.501-1.12 | 1.59E-01 | 1.269470 | 0.277-5.813 | 7.59E-01 | -4.12E-03 | 0.450 | 0.739928 | 7.15E-02 | NA | 0 |
| Bread type: Other type of bread | 22 | 16.334425 | 2.353-113.402 | 4.72E-03 | 138.200180 | 3.059-6.24E+03 | 1.97E-02 | -9.01E-03 | 0.226 | 14.600416 | 1.69E-03 | NA | 0 |
| Fresh fruit intake | 137 | 1.154370 | 0.875-1.523 | 3.10E-01 | 1.086622 | 0.464-2.543 | 8.48E-01 | 4.81E-04 | 0.889 | 1.147198 | 2.35E-01 | 0.331 | 2 |
| Dried fruit intake | 152 | 0.764697 | 0.625-0.936 | 9.12E-03 | 0.917734 | 0.476-1.769 | 7.98E-01 | -2.05E-03 | 0.561 | 0.738831 | 3.17E-04 | 0.709 | 1 |
| Salad / raw vegetable intake | 103 | 1.183824 | 0.888-1.579 | 2.50E-01 | 0.757362 | 0.337-1.702 | 5.03E-01 | 2.39E-03 | 0.535 | 0.953585 | 6.96E-01 | 0.879 | 3 |
| Cooked vegetable intake | 93 | 1.193186 | 0.901-1.58 | 2.18E-01 | 1.268456 | 0.611-2.635 | 5.25E-01 | -1.70E-04 | 0.963 | 1.247417 | 3.77E-02 | 0.816 | 1 |
| Age when last ate meat | 11 | 0.959803 | 0.847-1.087 | 5.18E-01 | 1.167375 | 0.831-1.64 | 3.95E-01 | -1.09E-02 | 0.438 | 1.020092 | 6.50E-01 | NA | 0 |
| Beef intake | 99 | 1.312602 | 1.024-1.682 | 3.17E-02 | 1.154765 | 0.681-1.959 | 5.95E-01 | 2.62E-03 | 0.376 | 1.383930 | 2.03E-04 | 0.641 | 1 |
| lamb/mutton intake | 123 | 1.108269 | 0.869-1.414 | 4.08E-01 | 0.930068 | 0.456-1.897 | 8.42E-01 | 1.68E-03 | 0.623 | 1.099433 | 3.82E-01 | 0.999 | 2 |
| pork intake | 81 | 1.403631 | 1.024-1.923 | 3.49E-02 | 0.897416 | 0.388-2.076 | 8.01E-01 | 5.14E-03 | 0.201 | 1.492214 | 4.24E-03 | 0.773 | 1 |
| Poultry intake | 81 | 1.307618 | 0.996-1.716 | 5.32E-02 | 1.734287 | 0.792-3.796 | 1.72E-01 | -3.26E-03 | 0.468 | 1.332518 | 1.39E-02 | NA | 0 |
| Processed meat intake | 124 | 0.993972 | 0.821-1.203 | 9.50E-01 | 0.752034 | 0.402-1.406 | 3.74E-01 | 3.55E-03 | 0.399 | 0.975897 | 7.57E-01 | 0.144 | 4 |
| Oily fish intake | 166 | 1.034662 | 0.877-1.221 | 6.87E-01 | 0.729133 | 0.431-1.234 | 2.41E-01 | 3.32E-03 | 0.322 | 0.965333 | 6.14E-01 | 0.862 | 1 |
| Non-oily fish intake | 62 | 0.970531 | 0.707-1.332 | 8.53E-01 | 0.949305 | 0.39-2.311 | 9.09E-01 | 1.34E-03 | 0.784 | 1.023788 | 8.53E-01 | 0.268 | 3 |
| Cheese intake | 201 | 0.711761 | 0.622-0.814 | 7.39E-07 | 0.626596 | 0.421-0.933 | 2.24E-02 | 1.95E-03 | 0.508 | 0.717375 | 5.56E-09 | 0.870 | 1 |
| Milk type used: Full cream | 27 | 0.995451 | 0.222-4.459 | 9.95E-01 | 1.203177 | 0.058-25.045 | 9.06E-01 | -1.28E-03 | 0.824 | 0.824062 | 7.15E-01 | 0.162 | 1 |
| Milk type used: Semi-skimmed | 14 | 0.274921 | 0.096-0.784 | 1.58E-02 | 0.085060 | 0.008-0.895 | 6.26E-02 | 1.13E-02 | 0.199 | 0.384690 | 6.41E-02 | NA | 0 |
| Milk type used: Skimmed | 26 | 2.434428 | 0.911-6.504 | 7.60E-02 | 1.441600 | 0.117-17.809 | 7.78E-01 | 2.43E-03 | 0.748 | 2.362055 | 4.64E-02 | 0.634 | 1 |
| Milk type used: Soya | 23 | 0.133111 | 0.017-1.072 | 5.81E-02 | 0.310273 | 0.002-59.805 | 6.67E-01 | -2.17E-03 | 0.797 | 0.142982 | 4.58E-03 | 0.876 | 2 |
| Milk type used: Other type of milk | 13 | 0.013665 | 0-1.472 | 7.22E-02 | 9.108001 | 0.003-3.30E+04 | 6.08E-01 | -5.90E-03 | 0.515 | 0.818373 | 9.26E-01 | NA | 0 |
| Milk type used: Never/rarely have milk | 22 | 1.101501 | 0.117-10.356 | 9.33E-01 | 7.773744 | 0.063-958.74 | 4.14E-01 | -5.15E-03 | 0.455 | 1.360246 | 7.31E-01 | NA | 0 |
| Coffee intake | 109 | 1.275480 | 0.971-1.675 | 8.03E-02 | 1.505512 | 0.968-2.341 | 7.20E-02 | -1.57E-03 | 0.537 | 1.327575 | 1.55E-03 | 0.803 | 1 |
| Coffee type: Decaffeinated coffee (any type) | 17 | 0.824296 | 0.317-2.144 | 6.92E-01 | 0.566615 | 0.092-3.478 | 5.49E-01 | 9.53E-03 | 0.230 | 1.658811 | 1.69E-01 | NA | 0 |
| Coffee type: Instant coffee | 29 | 1.121669 | 0.609-2.066 | 7.13E-01 | 2.292106 | 0.523-10.048 | 2.81E-01 | -8.21E-03 | 0.196 | 0.865415 | 5.39E-01 | 0.009 | 2 |
| Coffee type: Ground coffee (include espresso, filter etc) | 99 | 0.695726 | 0.477-1.015 | 5.94E-02 | 2.530979 | 0.772-8.294 | 1.28E-01 | -1.07E-02 | 0.012 | 0.568077 | 1.10E-03 | 0.554 | 2 |
| Coffee type: Other type of coffee | 6 | 9.369204 | 0.073-1.21E+03 | 3.67E-01 | 1.461072 | 0.001-2.41E+03 | 9.25E-01 | 3.40E-03 | 0.800 | 3.462933 | 5.27E-01 | NA | 0 |
| Tea intake | 137 | 1.209531 | 0.989-1.479 | 6.42E-02 | 1.126384 | 0.768-1.652 | 5.43E-01 | 1.02E-04 | 0.970 | 1.127653 | 6.07E-02 | 0.861 | 1 |
| Water intake | 156 | 1.139786 | 0.936-1.388 | 1.94E-01 | 0.848432 | 0.536-1.343 | 4.84E-01 | 3.66E-03 | 0.179 | 1.131626 | 8.95E-02 | NA | 0 |
| Hot drink temperature | 210 | 0.681588 | 0.535-0.869 | 1.98E-03 | 0.814340 | 0.376-1.764 | 6.03E-01 | -1.77E-03 | 0.551 | 0.663915 | 7.45E-05 | 0.845 | 1 |
| Alcohol usually taken with meals | 149 | 0.669491 | 0.513-0.874 | 3.17E-03 | 0.590367 | 0.243-1.436 | 2.47E-01 | 1.17E-03 | 0.744 | 0.705867 | 8.93E-04 | NA | 0 |
| Average weekly red wine intake | 94 | 0.806882 | 0.646-1.008 | 5.84E-02 | 0.914345 | 0.525-1.594 | 7.53E-01 | -1.65E-03 | 0.657 | 0.798036 | 7.38E-03 | 0.785 | 1 |
| Average weekly spirits intake | 43 | 1.123976 | 0.849-1.487 | 4.13E-01 | 1.081476 | 0.575-2.032 | 8.09E-01 | 2.40E-03 | 0.583 | 1.278597 | 7.58E-03 | NA | 0 |
| Average weekly fortified wine intake | 22 | 0.919220 | 0.422-2.001 | 8.32E-01 | 0.533199 | 0.138-2.063 | 3.73E-01 | 2.40E-03 | 0.583 | 1.059443 | 8.42E-01 | NA | 0 |
| Average weekly beer plus cider intake | 99 | 1.260928 | 0.96-1.656 | 9.55E-02 | 1.404174 | 0.706-2.791 | 3.35E-01 | -1.62E-04 | 0.966 | 1.354741 | 8.28E-03 | NA | 0 |
| Average weekly champagne plus white wine intake | 49 | 0.875462 | 0.659-1.164 | 3.60E-01 | 0.880105 | 0.414-1.873 | 7.42E-01 | -5.48E-04 | 0.910 | 0.844076 | 1.50E-01 | NA | 0 |
| Salt added to food | 249 | 1.117431 | 0.963-1.296 | 1.43E-01 | 1.189909 | 0.818 | 3.64E-01 | -2.39E-04 | 0.919 | 1.181785 | 5.63E-03 | 0.783 | 2 |

**Supplementary Table 6. Causality of genetically determined 45 dietary habits on Hip OA in sensitivity analysis.**

| **Exposure** | **N snps** | **Weighted median** | | | **MR Egger** | | | | | **MR Presso** | | | |
| --- | --- | --- | --- | --- | --- | --- | --- | --- | --- | --- | --- | --- | --- |
|  |  | **OR** | **95%CI** | **Pval** | **OR** | **95%CI** | **Pval** | **Intercept** | **Intercept**  **Pval** | **OR** | **Pval** | **Distortion**  **test** | **N outliers** |
| Cereal intake | 174 | 0.913481 | 0.73-1.142 | 4.28E-01 | 0.855261 | 0.453-1.615 | 6.30E-01 | 9.55E-04 | 0.798 | 0.930320 | 4.14E-01 | NA | 0 |
| Cereal type: Bran cereal (e.g. All Bran, Branflakes) | 13 | 1.115753 | 0.264-4.708 | 8.81E-01 | 4.611387 | 0.405-52.468 | 2.44E-01 | -4.83E-03 | 0.562 | 2.403883 | 7.10E-02 | NA | 0 |
| Cereal type: Biscuit cereal (e.g. Weetabix) | 26 | 0.671184 | 0.222-2.032 | 4.81E-01 | 0.177407 | 0.021-1.515 | 1.27E-01 | 5.20E-03 | 0.505 | 0.432696 | 8.74E-02 | 0.730 | 1 |
| Cereal type: Oat cereal (e.g. Ready Brek, porridge) | 22 | 1.443397 | 0.472-4.411 | 5.20E-01 | 0.497111 | 0.074-3.334 | 4.80E-01 | 8.76E-03 | 0.256 | 1.466119 | 3.58E-01 | NA | 0 |
| Cereal type: Muesli | 58 | 0.624531 | 0.322-1.21 | 1.63E-01 | 0.950794 | 0.209-4.335 | 9.48E-01 | -2.00E-03 | 0.687 | 0.729115 | 1.98E-01 | NA | 0 |
| Cereal type: Other (e.g. Cornflakes, Frosties) | 51 | 1.410840 | 0.673-2.958 | 3.62E-01 | 0.418822 | 0.074-2.366 | 3.29E-01 | 7.81E-03 | 0.187 | 1.285630 | 3.76E-01 | NA | 0 |
| Bread intake | 119 | 0.905953 | 0.711-1.154 | 4.23E-01 | 0.854100 | 0.457-1.595 | 6.22E-01 | 3.18E-04 | 0.940 | 0.869026 | 1.21E-01 | NA | 0 |
| Bread type: White | 119 | 0.868038 | 0.533-1.413 | 5.69E-01 | 0.531849 | 0.106-2.662 | 4.44E-01 | 2.11E-03 | 0.695 | 0.724974 | 1.15E-01 | NA | 0 |
| Bread type: Brown | 19 | 1.288253 | 0.259-6.419 | 7.57E-01 | 0.558877 | 0.025-12.655 | 7.19E-01 | 5.94E-03 | 0.475 | 1.637578 | 4.19E-01 | NA | 0 |
| Bread type: Wholemeal or wholegrain | 89 | 1.462625 | 0.87-2.458 | 1.51E-01 | 1.762338 | 0.23-13.507 | 5.87E-01 | -2.05E-03 | 0.778 | 1.368951 | 1.56E-01 | 0.917 | 2 |
| Bread type: Other type of bread | 22 | 1.466534 | 0.135-15.942 | 7.53E-01 | 9.275885 | 0.077-1.12E+03 | 3.73E-01 | -9.48E-03 | 0.312 | 0.885337 | 8.97E-01 | NA | 0 |
| Fresh fruit intake | 139 | 1.283784 | 0.881-1.87 | 1.93E-01 | 1.974534 | 0.683-5.704 | 2.11E-01 | -4.33E-03 | 0.319 | 1.281074 | 9.56E-02 | 0.998 | 2 |
| Dried fruit intake | 155 | 0.880366 | 0.687-1.127 | 3.13E-01 | 0.980452 | 0.45-2.135 | 9.60E-01 | -6.55E-05 | 0.987 | 0.960372 | 6.81E-01 | 0.854 | 1 |
| Salad / raw vegetable intake | 106 | 1.223987 | 0.851-1.76 | 2.75E-01 | 0.595925 | 0.245-1.45 | 2.57E-01 | 7.31E-03 | 0.088 | 1.245202 | 1.21E-01 | 0.822 | 1 |
| Cooked vegetable intake | 92 | 1.451068 | 1.026-2.053 | 3.55E-02 | 1.478188 | 0.61-3.584 | 3.89E-01 | -3.34E-04 | 0.940 | 1.425989 | 6.60E-03 | 0.824 | 2 |
| Age when last ate meat | 11 | 0.987548 | 0.823-1.185 | 8.93E-01 | 1.518791 | 0.974-2.367 | 9.80E-02 | -3.45E-02 | 0.081 | 0.991272 | 9.06E-01 | NA | 0 |
| Beef intake | 101 | 1.075804 | 0.775-1.494 | 6.63E-01 | 0.776181 | 0.314-1.921 | 5.85E-01 | 4.56E-03 | 0.369 | 1.087328 | 5.53E-01 | 0.803 | 1 |
| lamb/mutton intake | 128 | 0.681047 | 0.5-0.928 | 1.51E-02 | 0.632023 | 0.272-1.468 | 2.88E-01 | 2.72E-03 | 0.503 | 0.811860 | 1.06E-01 | 0.998 | 2 |
| pork intake | 81 | 1.366541 | 0.932-2.003 | 1.10E-01 | 1.732430 | 0.711-4.219 | 2.30E-01 | -2.68E-03 | 0.525 | 1.238074 | 1.46E-01 | NA | 0 |
| Poultry intake | 80 | 1.022421 | 0.737-1.419 | 8.95E-01 | 1.093157 | 0.486-2.459 | 8.30E-01 | -7.24E-04 | 0.876 | 1.049820 | 6.88E-01 | 0.197 | 2 |
| Processed meat intake | 129 | 0.965010 | 0.766-1.215 | 7.62E-01 | 0.780770 | 0.42-1.453 | 4.36E-01 | 2.22E-03 | 0.600 | 0.928639 | 3.86E-01 | NA | 0 |
| Oily fish intake | 169 | 1.255550 | 1.023-1.541 | 2.92E-02 | 0.847899 | 0.488-1.473 | 5.59E-01 | 4.62E-03 | 0.189 | 1.222421 | 7.30E-03 | NA | 0 |
| Non-oily fish intake | 67 | 1.245056 | 0.833-1.861 | 2.85E-01 | 0.929693 | 0.366-2.365 | 8.79E-01 | 3.80E-03 | 0.457 | 1.303970 | 9.25E-02 | NA | 0 |
| Cheese intake | 201 | 0.877886 | 0.744-1.036 | 1.24E-01 | 0.915067 | 0.569-1.471 | 7.15E-01 | -6.76E-04 | 0.847 | 0.861299 | 2.41E-02 | 0.859 | 1 |
| Milk type used: Full cream | 24 | 0.868714 | 0.098-7.737 | 9.00E-01 | 43.104265 | 0.365-5.09E+03 | 1.36E-01 | -1.44E-02 | 0.137 | 1.427145 | 6.97E-01 | 0.973 | 4 |
| Milk type used: Semi-skimmed | 13 | 1.429134 | 0.342-5.965 | 6.24E-01 | 4.588873 | 0.153-137.602 | 3.99E-01 | -1.05E-02 | 0.406 | 1.158930 | 8.33E-01 | 0.996 | 2 |
| Milk type used: Skimmed | 28 | 0.784484 | 0.251-2.451 | 6.76E-01 | 0.240305 | 0.011-5.417 | 3.78E-01 | 7.17E-03 | 0.451 | 0.841243 | 7.39E-01 | NA | 0 |
| Milk type used: Soya | 25 | 2.897803 | 0.274-30.65 | 3.77E-01 | 0.568190 | 0.002-159.833 | 8.46E-01 | 5.02E-03 | 0.570 | 2.656619 | 3.61E-01 | NA | 0 |
| Milk type used: Other type of milk | 11 | 73.894388 | 0.108-5.07E+04 | 1.97E-01 | 16.369221 | 0-1.49E+06 | 6.43E-01 | 2.50E-04 | 0.983 | 18.355844 | 2.90E-01 | 0.809 | 2 |
| Milk type used: Never/rarely have milk | 22 | 0.487133 | 0.03-8.012 | 6.15E-01 | 34.723593 | 0.141-8.57E+03 | 2.21E-01 | -1.26E-02 | 0.120 | 0.496330 | 5.04E-01 | NA | 0 |
| Coffee intake | 115 | 1.409173 | 1.079-1.841 | 1.19E-02 | 1.630655 | 1.022-2.601 | 4.24E-02 | -1.96E-03 | 0.497 | 1.409037 | 1.47E-03 | NA | 0 |
| Coffee type: Decaffeinated coffee (any type) | 17 | 1.046176 | 0.323-3.384 | 9.40E-01 | 0.313400 | 0.032-3.071 | 3.35E-01 | 8.13E-03 | 0.410 | 0.783142 | 5.64E-01 | NA | 0 |
| Coffee type: Instant coffee | 29 | 0.714643 | 0.349-1.463 | 3.58E-01 | 1.310004 | 0.249-6.89 | 7.52E-01 | -5.52E-03 | 0.442 | 0.612366 | 9.15E-02 | 0.656 | 1 |
| Coffee type: Ground coffee (include espresso, filter etc) | 99 | 0.710158 | 0.449-1.124 | 1.44E-01 | 2.288726 | 0.658-7.961 | 1.96E-01 | -6.81E-03 | 0.131 | 0.916858 | 6.39E-01 | NA | 0 |
| Coffee type: Other type of coffee | 6 | 8.355030 | 0.021-3.36E+03 | 4.88E-01 | 5.042588 | 0.001-3.58E+04 | 7.39E-01 | 6.00E-03 | 0.707 | 23.672286 | 1.39E-01 | NA | 0 |
| Tea intake | 138 | 1.339407 | 1.07-1.677 | 1.09E-02 | 1.553953 | 1.079-2.237 | 1.91E-02 | -3.53E-03 | 0.200 | 1.241149 | 3.41E-03 | 0.962 | 2 |
| Water intake | 158 | 1.013783 | 0.799-1.287 | 9.10E-01 | 0.611779 | 0.351-1.066 | 8.50E-02 | 8.30E-03 | 0.015 | 1.160685 | 1.11E-01 | 0.839 | 1 |
| Hot drink temperature | 211 | 0.621908 | 0.468-0.827 | 1.08E-03 | 1.133036 | 0.508-2.528 | 7.61E-01 | -3.92E-03 | 0.209 | 0.717986 | 2.33E-03 | NA | 0 |
| Alcohol usually taken with meals | 150 | 0.804211 | 0.576-1.123 | 2.01E-01 | 0.364002 | 0.127-1.047 | 6.28E-02 | 6.42E-03 | 0.135 | 0.810782 | 9.60E-02 | 0.949 | 2 |
| Average weekly red wine intake | 97 | 1.101611 | 0.846-1.434 | 4.72E-01 | 0.888611 | 0.457-1.727 | 7.28E-01 | 3.38E-03 | 0.442 | 1.143552 | 1.80E-01 | NA | 0 |
| Average weekly spirits intake | 43 | 1.465860 | 1.024-2.098 | 3.67E-02 | 0.842673 | 0.336-2.112 | 7.17E-01 | 5.83E-03 | 0.363 | 1.292414 | 8.70E-02 | NA | 0 |
| Average weekly fortified wine intake | 21 | 0.819787 | 0.31-2.171 | 6.89E-01 | 0.256481 | 0.048-1.373 | 1.28E-01 | 9.95E-03 | 0.132 | 0.873337 | 7.18E-01 | NA | 0 |
| Average weekly beer plus cider intake | 100 | 0.957518 | 0.698-1.313 | 7.88E-01 | 1.036543 | 0.482-2.231 | 9.27E-01 | -1.30E-03 | 0.762 | 0.898086 | 3.84E-01 | 0.850 | 1 |
| Average weekly champagne plus white wine intake | 48 | 0.701677 | 0.479-1.029 | 6.95E-02 | 1.460803 | 0.547-3.902 | 4.53E-01 | -9.20E-03 | 0.151 | 0.728005 | 4.72E-02 | NA | 0 |
| Salt added to food | 248 | 0.972031 | 0.818-1.155 | 7.47E-01 | 1.009974 | 0.686-1.486 | 9.60E-01 | -4.43E-05 | 0.985 | 0.995609 | 9.43E-01 | 0.040 | 1 |

**Supplementary Table 7. Causality of genetically determined 45 dietary habits on Spine OA in sensitivity analysis.**

| **Exposure** | **N snps** | **Weighted median** | | | **MR egger** | | | | | **MR Presso** | | | |
| --- | --- | --- | --- | --- | --- | --- | --- | --- | --- | --- | --- | --- | --- |
|  |  | **OR** | **95%CI** | **Pval** | **OR** | **95%CI** | **Pval** | **Intercept** | **Intercept**  **Pval** | **OR** | **Pval** | **Distortion test** | **N outliers** |
| Cereal intake | 174 | 0.863349 | 0.668-1.116 | 2.61E-01 | 0.828575 | 0.438-1.567 | 5.64E-01 | -1.18E-03 | 0.751749 | 0.737987 | 8.08E-04 | 0.837 | 1 |
| Cereal type: Bran cereal (e.g. All Bran, Branflakes) | 13 | 2.781410 | 0.517-14.955 | 2.33E-01 | 12.699483 | 0.76-212.138 | 1.05E-01 | -1.02E-02 | 0.303639 | 3.587297 | 3.98E-02 | NA | 0 |
| Cereal type: Biscuit cereal (e.g. Weetabix) | 27 | 1.072069 | 0.326-3.528 | 9.09E-01 | 0.623673 | 0.07-5.527 | 6.75E-01 | 6.33E-04 | 0.935725 | 0.736730 | 4.83E-01 | NA | 0 |
| Cereal type: Oat cereal (e.g. Ready Brek, porridge) | 23 | 0.923032 | 0.284-3.002 | 8.94E-01 | 0.327640 | 0.037-2.877 | 3.26E-01 | 1.28E-02 | 0.149446 | 1.432206 | 4.29E-01 | NA | 0 |
| Cereal type: Muesli | 59 | 0.294034 | 0.14-0.617 | 1.19E-03 | 0.697151 | 0.119-4.077 | 6.90E-01 | -5.10E-03 | 0.384509 | 0.324521 | 1.23E-04 | NA | 0 |
| Cereal type: Other (e.g. Cornflakes, Frosties) | 55 | 1.701197 | 0.753-3.845 | 2.01E-01 | 1.589430 | 0.218-11.566 | 6.49E-01 | 2.14E-03 | 0.747684 | 2.169484 | 1.66E-02 | 0.624 | 1 |
| Bread intake | 117 | 1.097009 | 0.836-1.439 | 5.04E-01 | 0.683573 | 0.353-1.322 | 2.61E-01 | 5.66E-03 | 0.20389 | 1.059018 | 5.62E-01 | 0.782 | 2 |
| Bread type: White | 122 | 2.172692 | 1.326-3.56 | 2.07E-03 | 1.443613 | 0.295-7.067 | 6.51E-01 | 2.66E-03 | 0.616065 | 2.047200 | 4.07E-04 | NA | 0 |
| Bread type: Brown | 20 | 0.411497 | 0.074-2.296 | 3.11E-01 | 1.229186 | 0.038-39.85 | 9.09E-01 | -4.02E-03 | 0.657762 | 0.583995 | 3.91E-01 | NA | 0 |
| Bread type: Wholemeal or wholegrain | 92 | 0.880171 | 0.519-1.494 | 6.36E-01 | 1.801760 | 0.252-12.869 | 5.59E-01 | -6.06E-03 | 0.393155 | 0.799990 | 3.00E-01 | NA | 0 |
| Bread type: Other type of bread | 22 | 1.906100 | 0.156-23.316 | 6.14E-01 | 5.447406 | 0.054-545.698 | 4.79E-01 | -4.61E-04 | 0.958779 | 4.861500 | 6.75E-02 | NA | 0 |
| Fresh fruit intake | 141 | 0.907120 | 0.602-1.366 | 6.41E-01 | 1.787847 | 0.635-5.03 | 2.73E-01 | -6.57E-03 | 0.119097 | 0.822291 | 1.77E-01 | 0.807 | 1 |
| Dried fruit intake | 156 | 0.687248 | 0.521-0.906 | 7.75E-03 | 1.045726 | 0.478-2.288 | 9.11E-01 | -3.96E-03 | 0.347654 | 0.736058 | 2.67E-03 | NA | 0 |
| Salad / raw vegetable intake | 108 | 1.061794 | 0.734-1.535 | 7.50E-01 | 1.412304 | 0.556-3.59 | 4.70E-01 | -4.04E-03 | 0.362321 | 0.889117 | 4.30E-01 | NA | 0 |
| Cooked vegetable intake | 94 | 1.257199 | 0.861-1.835 | 2.36E-01 | 2.530414 | 0.954-6.71 | 6.52E-02 | -6.70E-03 | 0.175155 | 1.320702 | 5.58E-02 | NA | 0 |
| Age when last ate meat | 11 | 1.031951 | 0.867-1.228 | 7.23E-01 | 1.006930 | 0.625-1.623 | 9.78E-01 | 6.10E-04 | 0.974848 | 1.014584 | 8.01E-01 | NA | 0 |
| Beef intake | 102 | 1.133247 | 0.816-1.574 | 4.56E-01 | 1.320270 | 0.605-2.883 | 4.87E-01 | -1.62E-03 | 0.713262 | 1.113509 | 3.73E-01 | 0.615 | 1 |
| lamb/mutton intake | 130 | 0.865681 | 0.612-1.225 | 4.15E-01 | 1.885675 | 0.781-4.554 | 1.61E-01 | -8.01E-03 | 0.060916 | 0.831807 | 1.82E-01 | NA | 0 |
| pork intake | 84 | 1.345073 | 0.869-2.083 | 1.84E-01 | 0.768341 | 0.282-2.097 | 6.08E-01 | 5.61E-03 | 0.240541 | 1.336698 | 7.96E-02 | NA | 0 |
| Poultry intake | 79 | 1.415669 | 0.966-2.076 | 7.50E-02 | 0.953014 | 0.342-2.655 | 9.27E-01 | 4.30E-03 | 0.463856 | 1.403359 | 2.80E-02 | NA | 0 |
| Processed meat intake | 129 | 0.858214 | 0.669-1.101 | 2.29E-01 | 0.893700 | 0.434-1.842 | 7.61E-01 | 7.88E-04 | 0.872501 | 0.965837 | 7.24E-01 | NA | 0 |
| Oily fish intake | 170 | 1.107774 | 0.89-1.379 | 3.59E-01 | 1.460199 | 0.823-2.591 | 1.97E-01 | -4.67E-03 | 0.204471 | 1.033615 | 6.64E-01 | NA | 0 |
| Non-oily fish intake | 66 | 0.797249 | 0.52-1.222 | 2.98E-01 | 1.616820 | 0.611-4.276 | 3.37E-01 | -6.27E-03 | 0.242042 | 0.972097 | 8.70E-01 | 0.945 | 2 |
| Cheese intake | 202 | 0.718569 | 0.601-0.859 | 2.73E-04 | 0.745727 | 0.474-1.173 | 2.06E-01 | -1.12E-03 | 0.738819 | 0.698861 | 2.43E-08 | NA | 0 |
| Milk type used: Full cream | 29 | 1.907326 | 0.294-12.371 | 4.98E-01 | 1.497327 | 0.035-63.214 | 8.34E-01 | -1.11E-04 | 0.987709 | 1.590619 | 4.71E-01 | NA | 0 |
| Milk type used: Semi-skimmed | 15 | 0.249322 | 0.059-1.048 | 5.79E-02 | 0.091148 | 0.003-3.156 | 2.08E-01 | 6.32E-03 | 0.625175 | 0.211386 | 3.11E-02 | NA | 0 |
| Milk type used: Skimmed | 30 | 1.196383 | 0.402-3.561 | 7.47E-01 | 1.569041 | 0.124-19.83 | 7.30E-01 | -1.14E-03 | 0.885903 | 1.368757 | 4.45E-01 | NA | 0 |
| Milk type used: Soya | 25 | 0.125573 | 0.009-1.807 | 1.27E-01 | 0.906014 | 0.005-162.228 | 9.71E-01 | -7.97E-04 | 0.922018 | 0.710661 | 6.96E-01 | NA | 0 |
| Milk type used: Other type of milk | 13 | 4.257144 | 0.011-1.64E+03 | 6.33E-01 | 168.42979 | 0.022-1.29E+06 | 2.85E-01 | -6.91E-03 | 0.484861 | 10.045794 | 1.76E-01 | NA | 0 |
| Milk type used: Never/rarely have milk | 21 | 2.117838 | 0.065-68.875 | 6.73E-01 | 0.435850 | 0-1.03E+03 | 8.36E-01 | -2.07E-04 | 0.984981 | 0.406221 | 5.29E-01 | NA | 0 |
| Coffee intake | 116 | 1.431534 | 1.036-1.978 | 2.97E-02 | 1.352306 | 0.836-2.188 | 2.22E-01 | -2.17E-03 | 0.467587 | 1.158949 | 1.78E-01 | NA | 0 |
| Coffee type: Decaffeinated coffee (any type) | 18 | 2.893554 | 0.762-10.99 | 1.19E-01 | 3.923231 | 0.27-56.913 | 3.31E-01 | -7.23E-03 | 0.547965 | 1.816580 | 2.73E-01 | NA | 0 |
| Coffee type: Instant coffee | 34 | 0.879618 | 0.383-2.019 | 7.62E-01 | 0.899270 | 0.112-7.191 | 9.21E-01 | -1.90E-03 | 0.828726 | 0.705218 | 2.63E-01 | NA | 0 |
| Coffee type: Ground coffee (include espresso, filter etc) | 105 | 0.692994 | 0.417-1.151 | 1.56E-01 | 1.998785 | 0.485-8.233 | 3.40E-01 | -9.12E-03 | 0.074363 | 0.565715 | 6.59E-03 | NA | 0 |
| Coffee type: Other type of coffee | 6 | 0.740962 | 0.001-809.999 | 9.33E-01 | 2.70E+03 | 0.212-3.43E+07 | 1.77E-01 | -3.62E-02 | 0.090791 | 0.325452 | 7.17E-01 | NA | 0 |
| Tea intake | 140 | 1.109916 | 0.887-1.39 | 3.63E-01 | 1.228667 | 0.822-1.836 | 3.17E-01 | -1.41E-03 | 0.64043 | 1.112437 | 1.94E-01 | 0.854 | 1 |
| Water intake | 161 | 1.071396 | 0.828-1.386 | 6.00E-01 | 0.770349 | 0.438-1.356 | 3.67E-01 | 4.10E-03 | 0.234438 | 1.062175 | 5.23E-01 | NA | 0 |
| Hot drink temperature | 213 | 0.648853 | 0.471-0.893 | 7.94E-03 | 1.028310 | 0.425-2.486 | 9.51E-01 | -3.20E-03 | 0.349519 | 0.696106 | 2.34E-03 | NA | 0 |
| Alcohol usually taken with meals | 153 | 0.720873 | 0.507-1.024 | 6.78E-02 | 0.455160 | 0.143-1.448 | 1.85E-01 | 3.38E-03 | 0.469987 | 0.698952 | 7.93E-03 | NA | 0 |
| Average weekly red wine intake | 98 | 0.815313 | 0.61-1.089 | 1.67E-01 | 0.990575 | 0.485-2.023 | 9.79E-01 | -2.93E-03 | 0.536802 | 0.803526 | 4.94E-02 | NA | 0 |
| Average weekly spirits intake | 42 | 1.392215 | 0.917-2.114 | 1.20E-01 | 1.050571 | 0.328-3.365 | 9.34E-01 | 3.06E-03 | 0.700567 | 1.369127 | 6.78E-02 | 0.534 | 2 |
| Average weekly fortified wine intake | 22 | 1.762450 | 0.592-5.245 | 3.08E-01 | 1.889173 | 0.182-19.623 | 6.00E-01 | -4.47E-03 | 0.612553 | 1.080047 | 8.76E-01 | NA | 0 |
| Average weekly beer plus cider intake | 101 | 0.791055 | 0.56-1.117 | 1.83E-01 | 0.650342 | 0.289-1.465 | 3.02E-01 | 5.52E-03 | 0.230946 | 1.039960 | 7.72E-01 | 0.348 | 1 |
| Average weekly champagne plus white wine intake | 49 | 0.653465 | 0.433-0.987 | 4.33E-02 | 0.919760 | 0.359-2.355 | 8.62E-01 | -4.45E-03 | 0.460255 | 0.654135 | 4.25E-03 | NA | 0 |
| Salt added to food | 250 | 1.123258 | 0.919-1.374 | 2.58E-01 | 0.981059 | 0.603-1.597 | 9.39E-01 | 2.17E-03 | 0.475782 | 1.165578 | 4.73E-02 | NA | 0 |

**Supplementary Table 8. Causality of genetically determined 45 dietary habits on Hand OA in sensitivity analysis.**

| **Exposure** | **N snps** | **Weighted median** | | | **MR egger** | | | | | **MR Presso** | | | |
| --- | --- | --- | --- | --- | --- | --- | --- | --- | --- | --- | --- | --- | --- |
|  |  | **OR** | **95%CI** | **Pval** | **OR** | **95%CI** | **Pval** | **Intercept** | **Intercept**  **Pval** | **OR** | **Pval** | **Distortion test** | **N outliers** |
| Cereal intake | 176 | 0.759894 | 0.562-1.028 | 7.51E-02 | 0.471723 | 0.214-1.038 | 6.36E-02 | 7.16E-03 | 0.122 | 0.903200 | 3.65E-01 | NA | 0 |
| Cereal type: Bran cereal (e.g. All Bran, Branflakes) | 12 | 9.161029 | 1.116-75.188 | 3.92E-02 | 4.550231 | 0.101-205.293 | 4.54E-01 | 7.18E-03 | 0.568 | 11.313153 | 8.28E-04 | NA | 0 |
| Cereal type: Biscuit cereal (e.g. Weetabix) | 27 | 1.555432 | 0.391-6.184 | 5.30E-01 | 1.440384 | 0.129-16.061 | 7.69E-01 | -1.28E-03 | 0.884 | 1.168355 | 7.21E-01 | NA | 0 |
| Cereal type: Oat cereal (e.g. Ready Brek, porridge) | 23 | 5.286995 | 1.174-23.802 | 3.01E-02 | 0.152672 | 0.01-2.269 | 1.87E-01 | 2.62E-02 | 0.022 | 3.723310 | 3.90E-02 | NA | 0 |
| Cereal type: Muesli | 59 | 0.847046 | 0.349-2.054 | 7.13E-01 | 1.035699 | 0.15-7.136 | 9.72E-01 | -2.56E-03 | 0.692 | 0.697955 | 2.49E-01 | NA | 0 |
| Cereal type: Other (e.g. Cornflakes, Frosties) | 54 | 1.705221 | 0.628-4.627 | 2.95E-01 | 4.345181 | 0.291-64.962 | 2.92E-01 | -7.69E-03 | 0.399 | 1.421328 | 4.06E-01 | NA | 0 |
| Bread intake | 119 | 1.127141 | 0.821-1.548 | 4.59E-01 | 0.739084 | 0.346-1.577 | 4.36E-01 | 5.42E-03 | 0.289 | 1.036733 | 7.52E-01 | NA | 0 |
| Bread type: White | 121 | 0.624093 | 0.32-1.218 | 1.67E-01 | 0.139096 | 0.016-1.181 | 7.32E-02 | 1.26E-02 | 0.080 | 0.900244 | 6.88E-01 | NA | 0 |
| Bread type: Brown | 20 | 1.293882 | 0.137-12.182 | 8.22E-01 | 0.009917 | 0-0.64 | 4.37E-02 | 2.74E-02 | 0.020 | 1.519376 | 6.03E-01 | NA | 0 |
| Bread type: Wholemeal or wholegrain | 93 | 0.885176 | 0.449-1.745 | 7.25E-01 | 4.810861 | 0.431-53.712 | 2.05E-01 | -1.18E-02 | 0.175 | 0.933077 | 7.90E-01 | NA | 0 |
| Bread type: Other type of bread | 22 | 12.235342 | 0.515-290.607 | 1.21E-01 | 3.69E+03 | 8.663-1.57E+06 | 1.51E-02 | -2.21E-02 | 0.064 | 13.272391 | 2.93E-02 | NA | 0 |
| Fresh fruit intake | 139 | 0.863931 | 0.533-1.399 | 5.52E-01 | 0.932810 | 0.291-2.995 | 9.07E-01 | 4.76E-04 | 0.921 | 1.011402 | 9.43E-01 | 0.041 | 2 |
| Dried fruit intake | 157 | 0.887785 | 0.637-1.237 | 4.82E-01 | 1.196204 | 0.455-3.144 | 7.17E-01 | -3.18E-03 | 0.540 | 0.903092 | 4.05E-01 | NA | 0 |
| Salad / raw vegetable intake | 109 | 0.978196 | 0.615-1.557 | 9.26E-01 | 1.252696 | 0.382-4.105 | 7.11E-01 | -9.75E-04 | 0.862 | 1.137840 | 4.77E-01 | NA | 0 |
| Cooked vegetable intake | 95 | 1.421184 | 0.917-2.202 | 1.15E-01 | 0.829420 | 0.274-2.509 | 7.41E-01 | 6.42E-03 | 0.250 | 1.588620 | 2.15E-03 | NA | 0 |
| Age when last ate meat | 11 | 1.039521 | 0.82-1.317 | 7.48E-01 | 1.251188 | 0.653-2.398 | 5.17E-01 | -1.36E-02 | 0.616 | 1.060463 | 5.27E-01 | NA | 0 |
| Beef intake | 101 | 0.852811 | 0.564-1.29 | 4.51E-01 | 0.274971 | 0.106-0.714 | 9.33E-03 | 1.17E-02 | 0.031 | 0.762204 | 6.76E-02 | 0.779 | 1 |
| lamb/mutton intake | 129 | 0.871373 | 0.574-1.323 | 5.18E-01 | 1.055425 | 0.381-2.927 | 9.18E-01 | -1.13E-03 | 0.819 | 0.930835 | 6.48E-01 | 0.894 | 1 |
| pork intake | 84 | 1.130186 | 0.677-1.886 | 6.40E-01 | 0.590190 | 0.16-2.179 | 4.31E-01 | 5.48E-03 | 0.374 | 1.036904 | 8.60E-01 | NA | 0 |
| Poultry intake | 81 | 1.315337 | 0.83-2.085 | 2.44E-01 | 0.616523 | 0.2-1.901 | 4.02E-01 | 7.52E-03 | 0.247 | 1.176260 | 3.40E-01 | 0.773 | 1 |
| Processed meat intake | 129 | 0.754011 | 0.557-1.021 | 6.78E-02 | 1.006517 | 0.43-2.357 | 9.88E-01 | -1.99E-03 | 0.730 | 0.853381 | 1.80E-01 | NA | 0 |
| Oily fish intake | 169 | 1.055523 | 0.813-1.37 | 6.84E-01 | 1.408901 | 0.729-2.722 | 3.09E-01 | -3.24E-03 | 0.443 | 1.073753 | 4.05E-01 | NA | 0 |
| Non-oily fish intake | 68 | 1.249823 | 0.758-2.062 | 3.83E-01 | 0.409344 | 0.128-1.311 | 1.37E-01 | 8.94E-03 | 0.167 | 0.894726 | 5.70E-01 | NA | 0 |
| Cheese intake | 203 | 1.027352 | 0.832-1.269 | 8.02E-01 | 0.996931 | 0.574-1.733 | 9.91E-01 | -7.93E-04 | 0.846 | 0.942744 | 4.38E-01 | NA | 0 |
| Milk type used: Full cream | 28 | 0.323678 | 0.028-3.689 | 3.64E-01 | 1.174714 | 0.006-237.447 | 9.53E-01 | -3.14E-03 | 0.759 | 0.441471 | 3.47E-01 | NA | 0 |
| Milk type used: Semi-skimmed | 15 | 1.982725 | 0.351-11.185 | 4.38E-01 | 0.436263 | 0.017-11.232 | 6.25E-01 | 7.30E-03 | 0.535 | 1.164390 | 7.99E-01 | NA | 0 |
| Milk type used: Skimmed | 28 | 1.153817 | 0.281-4.735 | 8.43E-01 | 1.520014 | 0.063-36.943 | 7.99E-01 | -3.91E-03 | 0.691 | 0.891098 | 8.19E-01 | 0.965 | 2 |
| Milk type used: Soya | 25 | 0.289645 | 0.011-7.379 | 4.53E-01 | 0.248705 | 0-125.038 | 6.65E-01 | 3.35E-03 | 0.733 | 0.687127 | 7.31E-01 | NA | 0 |
| Milk type used: Other type of milk | 12 | 4.137418 | 0.001-24377.637 | 7.49E-01 | 5.60E+04 | 0.208-1.51E+10 | 1.17E-01 | -3.06E-02 | 0.059 | 0.458459 | 8.37E-01 | NA | 0 |
| Milk type used: Never/rarely have milk | 22 | 0.422190 | 0.008-21.259 | 6.66E-01 | 0.108628 | 0-166.752 | 5.60E-01 | 5.41E-03 | 0.608 | 0.660509 | 7.37E-01 | NA | 0 |
| Coffee intake | 116 | 2.101394 | 1.4-3.154 | 3.39E-04 | 2.737758 | 1.661-4.513 | 1.36E-04 | -8.83E-03 | 0.005 | 1.415019 | 4.51E-03 | NA | 0 |
| Coffee type: Decaffeinated coffee (any type) | 18 | 1.694419 | 0.38-7.557 | 4.89E-01 | 1.795322 | 0.104-30.934 | 6.92E-01 | -1.69E-03 | 0.894 | 1.497043 | 4.71E-01 | NA | 0 |
| Coffee type: Instant coffee | 33 | 1.000722 | 0.374-2.675 | 9.99E-01 | 1.607047 | 0.106-24.302 | 7.34E-01 | -9.30E-03 | 0.422 | 0.529398 | 1.30E-01 | NA | 0 |
| Coffee type: Ground coffee (include espresso, filter etc) | 103 | 0.780104 | 0.42-1.449 | 4.32E-01 | 5.574780 | 1.19-26.115 | 3.15E-02 | -1.27E-02 | 0.024 | 1.111509 | 6.66E-01 | NA | 0 |
| Coffee type: Other type of coffee | 6 | 4.802705 | 0.001-24100.744 | 7.18E-01 | 0.252846 | 0-4.76E+04 | 8.35E-01 | 1.20E-02 | 0.576 | 6.157378 | 3.42E-01 | NA | 0 |
| Tea intake | 141 | 1.284336 | 0.986-1.672 | 6.31E-02 | 2.121718 | 1.343-3.353 | 1.59E-03 | -9.09E-03 | 0.009 | 1.199071 | 5.04E-02 | NA | 0 |
| Water intake | 160 | 0.963772 | 0.704-1.32 | 8.18E-01 | 0.456397 | 0.216-0.963 | 4.11E-02 | 9.92E-03 | 0.027 | 1.027869 | 8.13E-01 | 0.120 | 1 |
| Hot drink temperature | 208 | 0.852174 | 0.575-1.264 | 4.26E-01 | 0.854730 | 0.28-2.608 | 7.83E-01 | 2.13E-04 | 0.960 | 0.920625 | 5.79E-01 | 0.788 | 2 |
| Alcohol usually taken with meals | 153 | 0.850782 | 0.544-1.329 | 4.78E-01 | 1.426388 | 0.344-5.915 | 6.25E-01 | -4.01E-03 | 0.486 | 0.818744 | 2.35E-01 | NA | 0 |
| Average weekly red wine intake | 98 | 1.329041 | 0.933-1.893 | 1.15E-01 | 1.788070 | 0.739-4.328 | 2.01E-01 | -7.12E-03 | 0.225 | 1.093823 | 5.01E-01 | NA | 0 |
| Average weekly spirits intake | 44 | 1.752313 | 1.075-2.855 | 2.43E-02 | 1.125364 | 0.389-3.258 | 8.29E-01 | 4.03E-03 | 0.589 | 1.559073 | 1.16E-02 | NA | 0 |
| Average weekly fortified wine intake | 22 | 1.140086 | 0.326-3.992 | 8.38E-01 | 0.683033 | 0.06-7.724 | 7.61E-01 | 3.73E-03 | 0.680 | 1.097012 | 8.51E-01 | NA | 0 |
| Average weekly beer plus cider intake | 100 | 1.032274 | 0.699-1.525 | 8.73E-01 | 1.927622 | 0.81-4.589 | 1.41E-01 | -7.88E-03 | 0.110 | 0.945677 | 6.90E-01 | 0.257 | 2 |
| Average weekly champagne plus white wine intake | 49 | 0.967002 | 0.593-1.576 | 8.93E-01 | 1.491944 | 0.52-4.277 | 4.60E-01 | -6.81E-03 | 0.320 | 0.894157 | 5.12E-01 | NA | 0 |
| Salt added to food | 251 | 0.977306 | 0.771-1.238 | 8.49E-01 | 1.303946 | 0.769-2.212 | 3.26E-01 | -2.47E-03 | 0.454 | 1.080220 | 3.55E-01 | 0.875 | 1 |

**Supplementary Table 9. Causality of genetically determined 45 dietary habits on TKR in sensitivity analysis.**

| **Exposure** | **N snps** | **Weighted median** | | | **MR egger** | | | | | **MR Presso** | | | |
| --- | --- | --- | --- | --- | --- | --- | --- | --- | --- | --- | --- | --- | --- |
|  |  | **OR** | **95%CI** | **Pval** | **OR** | **95%CI** | **Pval** | **Intercept** | **Intercept**  **Pval** | **OR** | **Pval** | **Distortion test** | **N outliers** |
| Cereal intake | 173 | 0.721563 | 0.541-0.962 | 2.61E-02 | 0.742540 | 0.329-1.673 | 4.74E-01 | -6.91E-04 | 0.884 | 0.735711 | 7.02E-03 | 0.845 | 1 |
| Cereal type: Bran cereal (e.g. All Bran, Branflakes) | 13 | 2.000905 | 0.249-16.065 | 5.14E-01 | 20.290340 | 0.758-543.055 | 1.00E-01 | -1.56E-02 | 0.182 | 1.563884 | 5.42E-01 | NA | 0 |
| Cereal type: Biscuit cereal (e.g. Weetabix) | 26 | 0.884094 | 0.22-3.553 | 8.62E-01 | 0.973398 | 0.068-14.025 | 9.84E-01 | -3.27E-03 | 0.734 | 0.717416 | 5.37E-01 | 0.275 | 1 |
| Cereal type: Oat cereal (e.g. Ready Brek, porridge) | 22 | 0.988573 | 0.242-4.032 | 9.87E-01 | 1.165880 | 0.096-14.218 | 9.05E-01 | -2.43E-04 | 0.981 | 1.255325 | 6.67E-01 | NA | 0 |
| Cereal type: Muesli | 58 | 0.155491 | 0.062-0.387 | 6.37E-05 | 0.252601 | 0.032-1.972 | 1.95E-01 | -2.53E-03 | 0.709 | 0.180162 | 1.71E-06 | NA | 0 |
| Cereal type: Other (e.g. Cornflakes, Frosties) | 53 | 4.254306 | 1.474-12.279 | 7.42E-03 | 1.475971 | 0.118-18.405 | 7.64E-01 | 6.91E-03 | 0.421 | 3.984556 | 1.11E-03 | NA | 0 |
| Bread intake | 118 | 1.244465 | 0.899-1.722 | 1.87E-01 | 1.457939 | 0.61-3.483 | 3.98E-01 | -3.71E-03 | 0.527 | 1.037932 | 7.75E-01 | NA | 0 |
| Bread type: White | 117 | 1.301854 | 0.695-2.437 | 4.10E-01 | 0.806452 | 0.088-7.412 | 8.50E-01 | 4.70E-03 | 0.523 | 1.559046 | 1.06E-01 | 0.926 | 2 |
| Bread type: Brown | 20 | 4.955802 | 0.578-42.47 | 1.44E-01 | 0.042014 | 0.001-2.392 | 1.42E-01 | 2.26E-02 | 0.045 | 2.583634 | 2.71E-01 | NA | 0 |
| Bread type: Wholemeal or wholegrain | 87 | 0.594753 | 0.313-1.131 | 1.13E-01 | 0.603477 | 0.056-6.48 | 6.78E-01 | -1.75E-03 | 0.836 | 0.575552 | 2.75E-02 | 0.839 | 3 |
| Bread type: Other type of bread | 22 | 11.301107 | 0.513-248.901 | 1.24E-01 | 3.50E+02 | 0.898-1.36E+05 | 6.86E-02 | -1.50E-02 | 0.212 | 9.415111 | 7.51E-02 | NA | 0 |
| Fresh fruit intake | 139 | 0.994056 | 0.622-1.587 | 9.80E-01 | 2.808267 | 0.854-9.235 | 9.14E-02 | -7.12E-03 | 0.144 | 1.245804 | 2.01E-01 | 0.929 | 2 |
| Dried fruit intake | 157 | 0.610657 | 0.436-0.856 | 4.18E-03 | 0.748110 | 0.266-2.104 | 5.83E-01 | -1.70E-03 | 0.759 | 0.641924 | 1.09E-03 | NA | 0 |
| Salad / raw vegetable intake | 106 | 0.905110 | 0.565-1.45 | 6.79E-01 | 0.546732 | 0.172-1.741 | 3.09E-01 | 4.91E-03 | 0.374 | 0.871632 | 4.36E-01 | 0.983 | 2 |
| Cooked vegetable intake | 94 | 1.412142 | 0.874-2.281 | 1.58E-01 | 0.507943 | 0.124-2.074 | 3.48E-01 | 9.05E-03 | 0.201 | 1.213963 | 3.36E-01 | NA | 0 |
| Age when last ate meat | 11 | 0.902629 | 0.733-1.111 | 3.34E-01 | 1.382956 | 0.8-2.391 | 2.76E-01 | -3.03E-02 | 0.194 | 0.949882 | 5.27E-01 | NA | 0 |
| Beef intake | 101 | 1.817733 | 1.221-2.706 | 3.24E-03 | 1.041071 | 0.394-2.753 | 9.35E-01 | 7.29E-03 | 0.184 | 1.920901 | 2.87E-05 | NA | 0 |
| lamb/mutton intake | 127 | 1.553651 | 1.015-2.378 | 4.24E-02 | 0.765761 | 0.25-2.348 | 6.41E-01 | 5.87E-03 | 0.278 | 1.342523 | 8.42E-02 | 0.486 | 2 |
| pork intake | 83 | 1.295409 | 0.734-2.285 | 3.71E-01 | 1.635960 | 0.365-7.332 | 5.22E-01 | 1.32E-03 | 0.852 | 1.903187 | 7.99E-03 | NA | 0 |
| Poultry intake | 81 | 1.639148 | 1.041-2.581 | 3.28E-02 | 3.943170 | 1.224-12.706 | 2.42E-02 | -1.09E-02 | 0.105 | 1.556513 | 1.28E-02 | 0.760 | 1 |
| Processed meat intake | 128 | 1.056784 | 0.795-1.404 | 7.03E-01 | 0.733498 | 0.313-1.72 | 4.77E-01 | 5.83E-03 | 0.314 | 1.088145 | 4.69E-01 | 0.828 | 1 |
| Oily fish intake | 168 | 0.814807 | 0.625-1.062 | 1.29E-01 | 0.459016 | 0.21-1.005 | 5.32E-02 | 7.38E-03 | 0.140 | 0.830871 | 7.17E-02 | 0.861 | 1 |
| Non-oily fish intake | 66 | 0.920389 | 0.549-1.543 | 7.53E-01 | 0.707073 | 0.218-2.296 | 5.66E-01 | 4.99E-03 | 0.442 | 1.110605 | 5.98E-01 | NA | 0 |
| Cheese intake | 201 | 0.660330 | 0.531-0.821 | 1.88E-04 | 0.594937 | 0.334-1.059 | 7.92E-02 | 1.27E-03 | 0.767 | 0.660545 | 8.20E-07 | 0.844 | 1 |
| Milk type used: Full cream | 27 | 0.391803 | 0.042-3.629 | 4.09E-01 | 6.466084 | 0.032-1.30E+03 | 4.97E-01 | -5.55E-03 | 0.591 | 1.663737 | 5.84E-01 | 0.274 | 1 |
| Milk type used: Semi-skimmed | 15 | 0.514914 | 0.104-2.549 | 4.16E-01 | 0.295566 | 0.004-19.892 | 5.80E-01 | 6.60E-03 | 0.664 | 0.718812 | 6.69E-01 | NA | 0 |
| Milk type used: Skimmed | 28 | 1.848728 | 0.476-7.181 | 3.75E-01 | 0.202003 | 0.009-4.761 | 3.30E-01 | 1.02E-02 | 0.289 | 1.305912 | 6.37E-01 | 0.213 | 1 |
| Milk type used: Soya | 25 | 0.144710 | 0.005-3.887 | 2.50E-01 | 0.020724 | 0-12.877 | 2.50E-01 | 5.72E-03 | 0.573 | 0.117620 | 9.29E-02 | NA | 0 |
| Milk type used: Other type of milk | 13 | 0.005629 | 0-12.027 | 1.85E-01 | 5.408326 | 0-2.91E+05 | 7.67E-01 | -1.26E-02 | 0.301 | 0.030877 | 2.10E-01 | NA | 0 |
| Milk type used: Never/rarely have milk | 22 | 1.681974 | 0.027-103.465 | 8.05E-01 | 0.094140 | 0-2540.372 | 6.55E-01 | 3.41E-03 | 0.816 | 0.294958 | 5.26E-01 | NA | 0 |
| Coffee intake | 113 | 1.841784 | 1.148-2.954 | 1.13E-02 | 2.088342 | 1.034-4.217 | 4.24E-02 | -2.18E-03 | 0.588 | 1.746628 | 8.39E-05 | NA | 0 |
| Coffee type: Decaffeinated coffee (any type) | 18 | 5.068641 | 1.079-23.815 | 3.98E-02 | 1.283967 | 0.058-28.419 | 8.76E-01 | 1.05E-02 | 0.452 | 3.935697 | 3.93E-02 | NA | 0 |
| Coffee type: Instant coffee | 29 | 0.967469 | 0.363-2.581 | 9.47E-01 | 5.569724 | 0.541-57.291 | 1.60E-01 | -1.60E-02 | 0.118 | 0.832065 | 6.27E-01 | 0.211 | 1 |
| Coffee type: Ground coffee (include espresso, filter etc) | 104 | 0.336419 | 0.174-0.651 | 1.21E-03 | 2.925561 | 0.415-20.608 | 2.84E-01 | -1.49E-02 | 0.034 | 0.378980 | 6.87E-04 | NA | 0 |
| Coffee type: Other type of coffee | 6 | 1.087816 | 0-2392.782 | 9.83E-01 | 0.099765 | 0-1.36E+04 | 7.22E-01 | 2.38E-03 | 0.909 | 0.185953 | 3.85E-01 | NA | 0 |
| Tea intake | 139 | 1.614697 | 1.206-2.161 | 1.27E-03 | 2.281527 | 1.266-4.11 | 6.83E-03 | -8.56E-03 | 0.043 | 1.258996 | 2.20E-02 | NA | 0 |
| Water intake | 157 | 1.046659 | 0.772-1.419 | 7.69E-01 | 0.573964 | 0.287-1.149 | 1.19E-01 | 9.23E-03 | 0.026 | 1.224975 | 6.03E-02 | NA | 0 |
| Hot drink temperature | 207 | 0.585061 | 0.404-0.847 | 4.46E-03 | 0.625120 | 0.202-1.934 | 4.16E-01 | -3.59E-04 | 0.934 | 0.622700 | 1.73E-03 | 0.973 | 2 |
| Alcohol usually taken with meals | 151 | 0.586679 | 0.371-0.928 | 2.28E-02 | 0.286565 | 0.061-1.347 | 1.16E-01 | 5.38E-03 | 0.390 | 0.568999 | 2.15E-03 | NA | 0 |
| Average weekly red wine intake | 96 | 0.772942 | 0.549-1.088 | 1.40E-01 | 1.638269 | 0.695-3.861 | 2.62E-01 | -9.21E-03 | 0.109 | 0.850064 | 2.27E-01 | NA | 0 |
| Average weekly spirits intake | 43 | 1.210028 | 0.755-1.939 | 4.28E-01 | 0.551413 | 0.197-1.544 | 2.64E-01 | 1.03E-02 | 0.158 | 1.108129 | 5.30E-01 | NA | 0 |
| Average weekly fortified wine intake | 22 | 0.740093 | 0.212-2.586 | 6.37E-01 | 0.547800 | 0.058-5.152 | 6.04E-01 | 7.29E-03 | 0.389 | 1.376886 | 4.64E-01 | NA | 0 |
| Average weekly beer plus cider intake | 99 | 1.148170 | 0.742-1.776 | 5.35E-01 | 1.157804 | 0.416-3.226 | 7.80E-01 | -1.04E-04 | 0.985 | 1.137150 | 4.27E-01 | NA | 0 |
| Average weekly champagne plus white wine intake | 49 | 0.608519 | 0.372-0.994 | 4.74E-02 | 0.879387 | 0.243-3.181 | 8.46E-01 | -2.83E-03 | 0.732 | 0.709080 | 8.83E-02 | NA | 0 |
| Salt added to food | 248 | 1.288278 | 1.013-1.639 | 3.91E-02 | 1.308601 | 0.738-2.32 | 3.58E-01 | -1.11E-03 | 0.756 | 1.225098 | 3.00E-02 | NA | 0 |

**Supplementary Table 10. Causality of genetically determined 45 dietary habits on THR in sensitivity analysis.**

| **Exposure** | **N snps** | **Weighted median** | | | **MR egger** | | | | | **MR Presso** | | | |
| --- | --- | --- | --- | --- | --- | --- | --- | --- | --- | --- | --- | --- | --- |
|  |  | **OR** | **95%CI** | **Pval** | **OR** | **95%CI** | **Pval** | **Intercept** | **Intercept**  **Pval** | **OR** | **Pval** | **Distortion test** | **N outliers** |
| Cereal intake | 173 | 0.984740 | 0.759-1.277 | 9.08E-01 | 0.792696 | 0.392-1.604 | 5.19E-01 | 3.56E-03 | 0.389 | 1.065095 | 5.26E-01 | 0.855 | 1 |
| Cereal type: Bran cereal (e.g. All Bran, Branflakes) | 13 | 1.092900 | 0.196-6.107 | 9.19E-01 | 2.645484 | 0.137-51.028 | 5.33E-01 | -3.45E-03 | 0.734 | 1.446483 | 3.86E-01 | NA | 0 |
| Cereal type: Biscuit cereal (e.g. Weetabix) | 26 | 0.422541 | 0.115-1.554 | 1.95E-01 | 0.218029 | 0.014-3.478 | 2.92E-01 | 3.98E-03 | 0.687 | 0.455653 | 1.75E-01 | 0.558 | 1 |
| Cereal type: Oat cereal (e.g. Ready Brek, porridge) | 22 | 0.891154 | 0.244-3.253 | 8.62E-01 | 0.158216 | 0.013-1.95 | 1.66E-01 | 1.26E-02 | 0.218 | 0.745581 | 5.93E-01 | NA | 0 |
| Cereal type: Muesli | 58 | 0.852597 | 0.383-1.899 | 6.96E-01 | 1.554613 | 0.234-10.348 | 6.50E-01 | -4.47E-03 | 0.474 | 0.826587 | 5.24E-01 | NA | 0 |
| Cereal type: Other (e.g. Cornflakes, Frosties) | 53 | 1.433621 | 0.609-3.377 | 4.10E-01 | 0.410084 | 0.053-3.193 | 3.99E-01 | 9.14E-03 | 0.192 | 1.527790 | 2.04E-01 | NA | 0 |
| Bread intake | 118 | 0.887181 | 0.664-1.185 | 4.18E-01 | 0.967113 | 0.457-2.045 | 9.30E-01 | -1.26E-03 | 0.802 | 0.895879 | 3.16E-01 | NA | 0 |
| Bread type: White | 120 | 0.557407 | 0.316-0.983 | 4.33E-02 | 0.366579 | 0.053-2.518 | 3.09E-01 | 2.48E-03 | 0.698 | 0.500516 | 4.79E-03 | 0.771 | 1 |
| Bread type: Brown | 19 | 0.990083 | 0.136-7.211 | 9.92E-01 | 0.204301 | 0.005-7.914 | 4.06E-01 | 7.16E-03 | 0.462 | 0.748940 | 6.59E-01 | NA | 0 |
| Bread type: Wholemeal or wholegrain | 89 | 2.293979 | 1.227-4.289 | 9.31E-03 | 0.755253 | 0.057-10.034 | 8.32E-01 | 6.65E-03 | 0.472 | 2.005070 | 1.32E-02 | 0.761 | 3 |
| Bread type: Other type of bread | 22 | 1.082077 | 0.052-22.37 | 9.59E-01 | 5.802767 | 0.015-2317.451 | 5.71E-01 | -1.05E-02 | 0.380 | 0.461075 | 5.18E-01 | NA | 0 |
| Fresh fruit intake | 140 | 1.130535 | 0.734-1.741 | 5.78E-01 | 1.319864 | 0.403-4.319 | 6.47E-01 | -9.75E-04 | 0.839 | 1.274213 | 1.47E-01 | 0.722 | 1 |
| Dried fruit intake | 157 | 1.066248 | 0.79-1.439 | 6.75E-01 | 0.900239 | 0.375-2.159 | 8.14E-01 | 2.30E-03 | 0.624 | 1.089080 | 4.47E-01 | 0.694 | 1 |
| Salad / raw vegetable intake | 106 | 0.895794 | 0.588-1.366 | 6.09E-01 | 0.348256 | 0.118-1.032 | 5.97E-02 | 1.18E-02 | 0.025 | 1.171269 | 3.51E-01 | 0.194 | 3 |
| Cooked vegetable intake | 94 | 1.208641 | 0.8-1.825 | 3.68E-01 | 2.284942 | 0.778-6.71 | 1.36E-01 | -6.15E-03 | 0.257 | 1.267472 | 1.27E-01 | NA | 0 |
| Age when last ate meat | 11 | 1.019841 | 0.834-1.246 | 8.48E-01 | 1.478727 | 0.824-2.655 | 2.23E-01 | -3.18E-02 | 0.203 | 0.998304 | 9.85E-01 | NA | 0 |
| Beef intake | 100 | 1.073553 | 0.72-1.602 | 7.28E-01 | 0.801362 | 0.276-2.327 | 6.85E-01 | 5.62E-03 | 0.349 | 1.293635 | 1.21E-01 | 0.607 | 2 |
| lamb/mutton intake | 127 | 0.919336 | 0.625-1.353 | 6.69E-01 | 0.538457 | 0.209-1.386 | 2.02E-01 | 3.98E-03 | 0.381 | 0.784321 | 9.16E-02 | 0.335 | 2 |
| pork intake | 83 | 1.585445 | 1.008-2.493 | 4.60E-02 | 2.041570 | 0.671-6.211 | 2.12E-01 | -4.74E-03 | 0.369 | 1.205650 | 2.97E-01 | NA | 0 |
| Poultry intake | 81 | 0.907592 | 0.607-1.357 | 6.36E-01 | 0.743198 | 0.265-2.083 | 5.74E-01 | 1.99E-03 | 0.735 | 0.906732 | 5.29E-01 | NA | 0 |
| Processed meat intake | 127 | 0.942520 | 0.725-1.225 | 6.58E-01 | 0.729766 | 0.355-1.502 | 3.94E-01 | 4.09E-03 | 0.404 | 1.014463 | 8.83E-01 | 0.074 | 1 |
| Oily fish intake | 169 | 1.098462 | 0.866-1.394 | 4.40E-01 | 0.546857 | 0.262-1.143 | 1.10E-01 | 1.07E-02 | 0.023 | 1.272211 | 1.33E-02 | NA | 0 |
| Non-oily fish intake | 66 | 1.544131 | 0.966-2.469 | 6.97E-02 | 0.924852 | 0.32-2.67 | 8.86E-01 | 4.92E-03 | 0.399 | 1.464516 | 3.78E-02 | 0.746 | 1 |
| Cheese intake | 201 | 0.972403 | 0.796-1.188 | 7.84E-01 | 1.383798 | 0.777-2.465 | 2.72E-01 | -5.61E-03 | 0.190 | 0.931265 | 3.64E-01 | 0.869 | 1 |
| Milk type used: Full cream | 25 | 0.231279 | 0.021-2.534 | 2.31E-01 | 146.583371 | 0.782-27483.093 | 7.46E-02 | -2.03E-02 | 0.053 | 1.057533 | 9.57E-01 | 0.046 | 3 |
| Milk type used: Semi-skimmed | 14 | 2.156513 | 0.483-9.631 | 3.14E-01 | 8.766135 | 0.255-301.107 | 2.52E-01 | -1.02E-02 | 0.431 | 2.245356 | 2.51E-01 | 0.656 | 1 |
| Milk type used: Skimmed | 27 | 1.025653 | 0.27-3.894 | 9.70E-01 | 1.413674 | 0.033-60.494 | 8.58E-01 | -5.87E-03 | 0.610 | 0.627538 | 4.51E-01 | 0.289 | 2 |
| Milk type used: Soya | 25 | 1.930178 | 0.111-33.61 | 6.52E-01 | 1.908987 | 0.002-1520.978 | 8.51E-01 | 2.65E-03 | 0.801 | 4.279076 | 2.57E-01 | NA | 0 |
| Milk type used: Other type of milk | 12 | 404.668137 | 0.141-1.16E+06 | 1.39E-01 | 2.90E+03 | 0-23990414856 | 3.50E-01 | -6.74E-03 | 0.688 | 152.920932 | 2.11E-01 | 0.631 | 1 |
| Milk type used: Never/rarely have milk | 22 | 0.410716 | 0.009-17.903 | 6.44E-01 | 18.180982 | 0.007-44369.163 | 4.75E-01 | -9.38E-03 | 0.408 | 0.807616 | 8.88E-01 | NA | 0 |
| Coffee intake | 113 | 1.514207 | 1.104-2.077 | 1.00E-02 | 1.932807 | 1.159-3.224 | 1.30E-02 | -4.67E-03 | 0.142 | 1.327433 | 1.50E-02 | 0.752 | 1 |
| Coffee type: Decaffeinated coffee (any type) | 17 | 1.118745 | 0.285-4.396 | 8.72E-01 | 0.412807 | 0.026-6.506 | 5.39E-01 | 3.57E-03 | 0.762 | 0.617734 | 3.21E-01 | NA | 0 |
| Coffee type: Instant coffee | 29 | 0.488945 | 0.206-1.161 | 1.05E-01 | 0.560163 | 0.075-4.158 | 5.76E-01 | 3.06E-04 | 0.972 | 0.473217 | 4.33E-02 | 0.768 | 2 |
| Coffee type: Ground coffee (include espresso, filter etc) | 100 | 1.574084 | 0.895-2.767 | 1.15E-01 | 2.074657 | 0.465-9.25 | 3.41E-01 | -4.44E-03 | 0.408 | 1.139520 | 5.49E-01 | 0.191 | 2 |
| Coffee type: Other type of coffee | 6 | 4.839360 | 0.003-7.99E+03 | 6.77E-01 | 0.129029 | 0-5487.039 | 7.26E-01 | 1.67E-02 | 0.392 | 10.835026 | 3.87E-01 | NA | 0 |
| Tea intake | 139 | 1.462433 | 1.116-1.917 | 5.87E-03 | 1.894953 | 1.239-2.899 | 3.78E-03 | -8.24E-03 | 0.011 | 1.129483 | 1.61E-01 | 0.811 | 1 |
| Water intake | 159 | 1.261933 | 0.953-1.671 | 1.04E-01 | 0.606807 | 0.321-1.146 | 1.26E-01 | 1.01E-02 | 0.010 | 1.355489 | 6.19E-03 | NA | 0 |
| Hot drink temperature | 210 | 0.603358 | 0.424-0.859 | 5.06E-03 | 0.804417 | 0.296-2.183 | 6.70E-01 | -1.74E-03 | 0.653 | 0.654258 | 1.26E-03 | NA | 0 |
| Alcohol usually taken with meals | 150 | 0.866880 | 0.586-1.282 | 4.75E-01 | 0.171540 | 0.042-0.694 | 1.46E-02 | 1.39E-02 | 0.015 | 0.969029 | 8.50E-01 | 0.338 | 1 |
| Average weekly red wine intake | 97 | 1.289232 | 0.945-1.758 | 1.08E-01 | 1.162400 | 0.527-2.564 | 7.10E-01 | 1.17E-03 | 0.823 | 1.264120 | 5.06E-02 | NA | 0 |
| Average weekly spirits intake | 42 | 1.280999 | 0.829-1.98 | 2.65E-01 | 0.422590 | 0.157-1.139 | 9.64E-02 | 1.43E-02 | 0.045 | 1.182122 | 3.26E-01 | NA | 0 |
| Average weekly fortified wine intake | 21 | 0.621000 | 0.178-2.161 | 4.54E-01 | 0.063095 | 0.006-0.67 | 3.35E-02 | 1.94E-02 | 0.041 | 0.706447 | 5.22E-01 | NA | 0 |
| Average weekly beer plus cider intake | 99 | 1.199277 | 0.825-1.744 | 3.41E-01 | 1.090986 | 0.434-2.745 | 8.54E-01 | -1.61E-03 | 0.755 | 0.918246 | 5.60E-01 | NA | 0 |
| Average weekly champagne plus white wine intake | 48 | 0.735752 | 0.457-1.184 | 2.06E-01 | 2.059669 | 0.662-6.404 | 2.18E-01 | -1.51E-02 | 0.042 | 0.652535 | 2.54E-02 | 0.712 | 1 |
| Salt added to food | 250 | 0.984734 | 0.799-1.213 | 8.85E-01 | 0.992493 | 0.632-1.559 | 9.74E-01 | 1.17E-04 | 0.967 | 0.979841 | 7.81E-01 | NA | 0 |

**Supplementary Table 11. FDR results of associations between dietary habits and** **subtypes related to osteoarthritis.**

| **Exposure** | **Knee OA** | **Hip OA** | **Spine OA** | **Hand OA** | **TKR** | **THR** |
| --- | --- | --- | --- | --- | --- | --- |
| Cereal intake | 1.35E-03 | 6.30E-01 | 1.54E-02 | 4.11E-01 | 1.83E-02 | 7.12E-01 |
| Cereal type: Bran cereal (e.g. All Bran, Branflakes) | 5.03E-01 | 3.00E-01 | 1.83E-01 | 1.52E-02 | 4.83E-01 | 6.61E-01 |
| Cereal type: Biscuit cereal (e.g. Weetabix) | 8.35E-01 | 3.65E-02 | 6.08E-01 | 8.35E-01 | 6.08E-01 | 1.17E-01 |
| Cereal type: Oat cereal (e.g. Ready Brek, porridge) | 8.78E-01 | 6.30E-01 | 6.03E-01 | 8.09E-02 | 8.78E-01 | 6.35E-01 |
| Cereal type: Muesli | 1.14E-04 | 3.39E-01 | 5.90E-04 | 5.14E-01 | 1.14E-06 | 6.35E-01 |
| Cereal type: Other (e.g. Cornflakes, Frosties) | 6.75E-04 | 5.80E-01 | 3.25E-02 | 6.30E-01 | 8.52E-03 | 3.61E-01 |
| Bread intake | 9.34E-01 | 3.59E-01 | 8.47E-01 | 6.30E-01 | 6.30E-01 | 5.11E-01 |
| Bread type: White | 1.48E-01 | 3.14E-01 | 2.48E-03 | 8.36E-01 | 2.17E-01 | 4.89E-02 |
| Bread type: Brown | 4.41E-01 | 6.30E-01 | 6.30E-01 | 7.55E-01 | 4.41E-01 | 8.28E-01 |
| Bread type: Wholemeal or wholegrain | 1.56E-01 | 4.41E-01 | 4.92E-01 | 8.74E-01 | 2.43E-02 | 8.99E-02 |
| Bread type: Other type of bread | 6.75E-04 | 9.15E-01 | 2.18E-01 | 9.77E-02 | 1.29E-01 | 6.30E-01 |
| Fresh fruit intake | 4.78E-01 | 5.59E-01 | 3.61E-01 | 9.55E-01 | 5.70E-01 | 6.07E-01 |
| Dried fruit intake | 1.54E-02 | 8.78E-01 | 1.75E-02 | 6.09E-01 | 1.16E-02 | 6.07E-01 |
| Salad / raw vegetable intake | 8.78E-01 | 2.95E-01 | 7.61E-01 | 7.04E-01 | 7.52E-01 | 6.30E-01 |
| Cooked vegetable intake | 1.46E-01 | 1.97E-02 | 1.36E-01 | 3.63E-02 | 5.59E-01 | 3.00E-01 |
| Age when last ate meat | 8.28E-01 | 9.15E-01 | 8.90E-01 | 6.85E-01 | 7.11E-01 | 9.83E-01 |
| Beef intake | 4.48E-04 | 5.83E-01 | 4.73E-01 | 1.67E-01 | 6.01E-04 | 3.21E-01 |
| lamb/mutton intake | 6.17E-01 | 3.62E-01 | 4.18E-01 | 8.39E-01 | 1.81E-01 | 3.45E-01 |
| pork intake | 2.06E-02 | 1.51E-01 | 1.83E-01 | 9.03E-01 | 4.89E-02 | 3.61E-01 |
| Poultry intake | 9.28E-02 | 8.78E-01 | 1.51E-01 | 5.70E-01 | 7.20E-02 | 6.32E-01 |
| Processed meat intake | 8.73E-01 | 5.83E-01 | 7.52E-01 | 5.04E-01 | 6.08E-01 | 9.01E-01 |
| Oily fish intake | 6.30E-01 | 4.99E-02 | 8.78E-01 | 5.49E-01 | 1.63E-01 | 8.09E-02 |
| Non-oily fish intake | 8.19E-01 | 2.55E-01 | 7.79E-01 | 7.20E-01 | 7.61E-01 | 1.17E-01 |
| Cheese intake | 1.20E-07 | 1.53E-01 | 1.20E-07 | 6.48E-01 | 5.19E-06 | 7.36E-01 |
| Milk type used: Full cream | 8.74E-01 | 8.71E-01 | 7.52E-01 | 6.59E-01 | 7.48E-01 | 9.63E-01 |
| Milk type used: Semi-skimmed | 5.62E-02 | 8.90E-01 | 1.83E-02 | 8.78E-01 | 7.52E-01 | 3.61E-01 |
| Milk type used: Skimmed | 1.83E-01 | 7.55E-01 | 6.85E-01 | 8.28E-01 | 9.34E-01 | 5.92E-01 |
| Milk type used: Soya | 2.17E-01 | 5.20E-01 | 8.47E-01 | 8.67E-01 | 2.00E-01 | 3.77E-01 |
| Milk type used: Other type of milk | 9.34E-01 | 4.45E-01 | 5.90E-01 | 8.78E-01 | 4.59E-01 | 4.11E-01 |
| Milk type used: Never/rarely have milk | 8.39E-01 | 7.03E-01 | 6.56E-01 | 8.73E-01 | 7.11E-01 | 9.07E-01 |
| Coffee intake | 1.54E-02 | 1.67E-02 | 4.40E-01 | 1.43E-02 | 1.35E-03 | 5.17E-02 |
| Coffee type: Decaffeinated coffee (any type) | 3.45E-01 | 7.52E-01 | 4.11E-01 | 6.79E-01 | 6.12E-02 | 6.08E-01 |
| Coffee type: Instant coffee | 7.52E-01 | 4.07E-01 | 4.59E-01 | 3.61E-01 | 8.67E-01 | 2.38E-01 |
| Coffee type: Ground coffee (include espresso, filter etc) | 1.80E-02 | 7.66E-01 | 4.89E-02 | 9.83E-01 | 8.52E-03 | 7.52E-01 |
| Coffee type: Other type of coffee | 7.07E-01 | 4.11E-01 | 8.19E-01 | 7.52E-01 | 7.55E-01 | 6.30E-01 |
| Tea intake | 1.63E-01 | 1.96E-02 | 3.45E-01 | 1.63E-01 | 6.60E-02 | 3.61E-01 |
| Water intake | 1.83E-01 | 2.50E-01 | 7.07E-01 | 8.78E-01 | 1.51E-01 | 4.70E-02 |
| Hot drink temperature | 6.18E-04 | 8.52E-03 | 1.54E-02 | 6.25E-01 | 7.91E-03 | 1.27E-02 |
| Alcohol usually taken with meals | 4.31E-03 | 2.24E-01 | 4.23E-02 | 6.30E-01 | 1.54E-02 | 8.47E-01 |
| Average weekly red wine intake | 8.26E-02 | 4.18E-01 | 1.57E-01 | 8.28E-01 | 4.10E-01 | 1.67E-01 |
| Average weekly spirits intake | 6.60E-02 | 1.83E-01 | 1.83E-01 | 8.09E-02 | 6.85E-01 | 6.30E-01 |
| Average weekly fortified wine intake | 8.90E-01 | 8.35E-01 | 9.15E-01 | 8.95E-01 | 6.95E-01 | 6.30E-01 |
| Average weekly beer plus cider intake | 2.79E-02 | 7.14E-01 | 8.67E-01 | 9.15E-01 | 6.30E-01 | 8.44E-01 |
| Average weekly champagne plus white wine intake | 2.49E-01 | 1.53E-01 | 2.21E-02 | 7.11E-01 | 1.55E-01 | 9.28E-02 |
| Salt added to food | 5.17E-02 | 9.34E-01 | 1.79E-01 | 6.30E-01 | 1.63E-01 | 9.83E-01 |

**Supplementary Table 12. MVMR analysis in 23 reliable causality evidence. (M1: Controlling for BMI; M2:** **Controlling for TB-BMD; M3:** **Controlling for Ever smoked)**

| **Exposure** | **Outcome** | **M1** | | **M2** | | **M3** | |  |
| --- | --- | --- | --- | --- | --- | --- | --- | --- |
|  |  | **IVW-Pval** | **Intercept Pval** | **IVW-Pval** | **Intercept Pval** | **IVW-Pval** | **Intercept Pval** | |
| Cereal intake | Knee OA | 8.92E-02 | 0.291 | 5.53E-04 | 0.813 | 5.10E-05 | 0.225 | |
| Cereal type: Muesli | Knee OA | 4.39E-03 | 0.681 | 2.14E-03 | 0.685 | 1.46E-05 | 0.705 | |
| Dried fruit intake | Knee OA | 3.37E-02 | 0.488 | 6.44E-05 | 0.779 | 3.28E-03 | 0.254 | |
| Beef intake | Knee OA | 1.80E-01 | 0.763 | 2.17E-03 | 0.358 | 3.49E-02 | 0.82 | |
| Cheese intake | Knee OA | 4.33E-02 | 0.994 | 9.39E-08 | 0.51 | 4.69E-08 | 0.468 | |
| Hot drink temperature | Knee OA | 4.72E-03 | 0.834 | 6.91E-05 | 0.845 | 7.35E-03 | 0.031 | |
| Alcohol usually taken with meals | Knee OA | 7.78E-02 | 0.116 | 1.29E-02 | 0.646 | 1.24E-02 | 0.387 | |
| Cooked vegetable intake | Hip OA | 3.00E-01 | 0.446 | 1.08E-02 | 0.379 | 5.71E-03 | 0.862 | |
| Coffee intake | Hip OA | 1.89E-03 | 0.921 | 2.30E-03 | 0.741 | 3.96E-04 | 0.007 | |
| Tea intake | Hip OA | 3.49E-01 | 0.588 | 4.39E-02 | 0.357 | 3.92E-03 | 0.051 | |
| Cereal type: Muesli | Spine OA | 7.61E-04 | 0.209 | 1.34E-02 | 0.962 | 1.05E-02 | 0.285 | |
| Cheese intake | Spine OA | 2.34E-02 | 0.804 | 3.06E-11 | 0.37 | 1.35E-06 | 0.115 | |
| Average weekly champagne plus white wine intake | Spine OA | 2.10E-01 | 0.058 | 2.23E-03 | 0.25 | 1.23E-02 | 0.759 | |
| Cereal intake | TKR | 5.07E-01 | 0.552 | 8.15E-04 | 0.685 | 3.13E-03 | 0.11 | |
| Cereal type: Muesli | TKR | 3.49E-04 | 0.396 | 9.58E-05 | 0.303 | 8.04E-05 | 0.693 | |
| Dried fruit intake | TKR | 4.67E-02 | 0.782 | 5.53E-04 | 0.95 | 4.96E-03 | 0.652 | |
| Beef intake | TKR | 3.08E-01 | 0.416 | 4.36E-05 | 0.644 | 9.43E-03 | 0.964 | |
| Cheese intake | TKR | 2.65E-01 | 0.856 | 9.00E-05 | 0.11 | 6.52E-06 | 0.489 | |
| Coffee intake | TKR | 2.55E-01 | 0.257 | 3.77E-03 | 0.666 | 1.92E-04 | 0.001 | |
| Hot drink temperature | TKR | 2.30E-01 | 0.126 | 2.84E-03 | 0.737 | 3.19E-03 | 0.109 | |
| Alcohol usually taken with meals | TKR | 2.79E-02 | 0.269 | 3.24E-02 | 0.688 | 5.12E-03 | 0.232 | |
| Bread type: White | THR | 1.73E-03 | 0.126 | 1.86E-01 | 0.901 | 4.08E-01 | 0.633 | |
| Hot drink temperature | THR | 4.99E-03 | 0.971 | 2.13E-03 | 0.929 | 3.75E-02 | 0.045 | |

**Supplementary Table 13. Results of LDSC for all FDR-corrected positive results in univariate analysis.**

| **Level of Evidence** | **Phenotype 1** | **Phenotype 2** | **Rg** | **Se** | **Pval** |
| --- | --- | --- | --- | --- | --- |
| Reliable evidence | Cereal intake | Knee OA | -0.156 | 0.033 | 1.83E-06 |
|  | Cereal type: Muesli | Knee OA | -0.277 | 0.037 | 4.48E-14 |
|  | Dried fruit intake | Knee OA | -0.155 | 0.032 | 1.64E-06 |
|  | Cheese intake | Knee OA | -0.208 | 0.029 | 9.67E-13 |
|  | Hot drink temperature | Knee OA | -0.095 | 0.031 | 1.80E-03 |
|  | Alcohol usually taken with meals | Knee OA | -0.181 | 0.032 | 9.61E-09 |
|  | Beef intake | Knee OA | 0.184 | 0.035 | 1.15E-07 |
|  | Cooked vegetable intake | Hip OA | 0.117 | 0.039 | 2.67E-03 |
|  | Coffee intake | Hip OA | 0.100 | 0.040 | 1.19E-02 |
|  | Tea intake | Hip OA | 0.038 | 0.037 | 2.98E-01 |
|  | Cereal type: Muesli | Spine OA | -0.331 | 0.048 | 5.87E-12 |
|  | Cheese intake | Spine OA | -0.327 | 0.046 | 7.73E-13 |
|  | Average weekly champagne plus white wine intake | Spine OA | -0.197 | 0.068 | 3.74E-03 |
|  | Cereal intake | TKR | -0.210 | 0.037 | 9.54E-09 |
|  | Cereal type: Muesli | TKR | -0.307 | 0.042 | 1.60E-13 |
|  | Dried fruit intake | TKR | -0.212 | 0.037 | 7.79E-09 |
|  | Cheese intake | TKR | -0.203 | 0.035 | 6.59E-09 |
|  | Hot drink temperature | TKR | -0.112 | 0.035 | 1.44E-03 |
|  | Alcohol usually taken with meals | TKR | -0.206 | 0.034 | 1.15E-09 |
|  | Beef intake | TKR | 0.197 | 0.041 | 1.40E-06 |
|  | Coffee intake | TKR | 0.165 | 0.044 | 1.95E-04 |
|  | Bread type: White | THR | -0.044 | 0.153 | 7.75E-01 |
|  | Hot drink temperature | THR | 0.002 | 0.109 | 9.89E-01 |
| Insufficient evidence | Coffee intake | Knee OA | 0.152 | 0.038 | 6.49E-05 |
|  | Average weekly beer plus cider intake | Knee OA | 0.092 | 0.034 | 6.08E-03 |
|  | Salt added to food | Knee OA | 0.119 | 0.028 | 2.31E-05 |
|  | Cereal intake | Spine OA | -0.187 | 0.044 | 2.22E-05 |
|  | Milk type used: Semi-skimmed | Spine OA | -0.305 | 0.076 | 5.43E-05 |
|  | Alcohol usually taken with meals | Spine OA | -0.201 | 0.046 | 1.22E-05 |
|  | Bread type: Wholemeal or wholegrain | TKR | -0.164 | 0.043 | 1.26E-04 |
|  | pork intake | TKR | 0.175 | 0.044 | 8.19E-05 |
| Weak evidence | Biscuit cereal | Hip OA | 0.003 | 0.075 | 9.64E-01 |
